# Dendritic geometry shapes neuronal cAMP signalling to the nucleus

**DOI:** 10.1038/ncomms7319

**Published:** 2015-02-18

**Authors:** Lu Li, Nicolas Gervasi, Jean-Antoine Girault

**Affiliations:** 1 Inserm, UMR-S 839, 75005 Paris, France; 2 Université Pierre et Marie Curie (UPMC, Paris 6), Sorbonne Universités, 75005 Paris, France; 3 Institut du Fer à Moulin, 75005 Paris, France

## Abstract

Neurons have complex dendritic trees, receiving numerous inputs at various distances from the cell body. Yet the rules of molecular signal propagation from dendrites to nuclei are unknown. DARPP-32 is a phosphorylation-regulated signalling hub in striatal output neurons. We combine diffusion-reaction modelling and live imaging to investigate cAMP-activated DARPP-32 signalling to the nucleus. The model predicts maximal effects on the nucleus of cAMP production in secondary dendrites, due to segmental decrease of dendrite diameter. Variations in branching, perikaryon size or spines have less pronounced effects. Biosensor kinase activity measurement following cAMP or dopamine uncaging confirms these predictions. Histone 3 phosphorylation, regulated by this pathway, is best stimulated by cAMP released in secondary-like dendrites. Thus, unexpectedly, the efficacy of diffusion-based signalling from dendrites to nucleus is not inversely proportional to the distance. We suggest a general mechanism by which dendritic geometry counterbalances the effect of dendritic distance for signalling to the nucleus.

## Abstract (web summary)

Neurons have complex dendritic trees but the rules governing the propagation of signals from dendrites to nuclei remain unclear. Here the authors combine diffusion-reaction modelling and live imaging to investigate the mechanisms regulating cAMP signalling in neurons and find that dendritic tree geometry shapes synapse-to-nucleus signalling.

## Graphical abstract

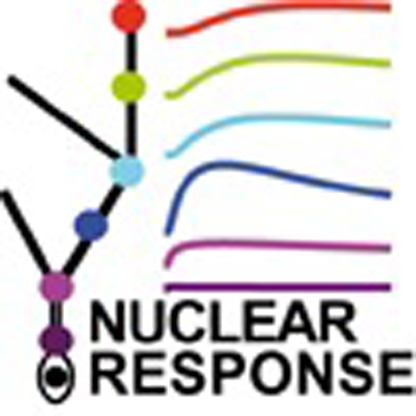

Dendrites are the main recipients of synaptic and neuromodulatory inputs. Their membrane properties and elaborate morphologies determine the input–output functions^1^. Synaptic integration depends on dendrites morphology, and current flow attenuation can be compensated by dendrites active properties^2^,^3^. Synaptic activation also triggers signalling pathways that regulate transcription and epigenetic responses in the nucleus, a critical step for long-term plasticity^4^,^5^,^6^. However, little is known about the propagation and integration of these molecular signals in dendrites. Biophysical studies have shown that rules governing Ca^2+^ diffusion, extrusion and buffering present formal similarities with cable equations for passive electrical propagation^7^,^8^, but these approaches have not been extended to more complex signalling pathways. Modelling and numerical simulations outlined the general importance of cell shape, a feature particularly important in neurons^9^,^10^. The use of biosensors showed how the signalling pathway activation can spread locally within microdomains^11^,^12^,^13^, and how the activation of a few excitatory spines can trigger wider ERK activity, including in the nucleus^14^. Yet the rules that may govern the spreading of biochemical signals from dendrites to nucleus are not known.

To understand the relationship between the dendrite morphology and signalling to the nucleus we focused on cyclic AMP (cAMP)-dependent signalling in striatal medium-size spiny neurons (MSNs), which is activated by several receptors including D1 dopamine receptors (D1R). It mediates phasic effects of dopamine in action selection, movement control and motivation, and is critical in dysfunctions of the basal ganglia including drug addiction and L-DOPA-induced dyskinesia in Parkinson’s disease^15^. We investigated the spatiotemporal dynamics of the pathway involving cAMP, cAMP-dependent protein kinase (PKA) and 32-kDa dopamine- and cAMP-regulated phosphoprotein (DARPP-32). DARPP-32 is a hub for signalling by multiple receptors^16^,^17^ and its regulation has been modelled by several groups^18^,^19^,^20^,^21^,^22^. PKA phosphorylates DARPP-32 at Thr34, turning it into a potent inhibitor of serine/threonine protein phosphatase-1 (PP1)^23^. DARPP-32 can also convey information from the cytoplasm to the nucleus where it contributes to chromatin modification and long-term changes^24^. To address the rules of this dendrite-to-nucleus signalling, we combined computational modelling and live cell imaging. Our models and experimental data reveal an unexpected relationship between the distance of stimuli from the soma and nuclear PKA activity. Our results show that dendritic morphology and biochemical properties of signalling pathways shape signalling from synapses to nucleus and can counterbalance the effects of distance. These observations result from simple principles likely to have implications for other pathways and neurons.

## Results

### DARPP-32 moves in dendrites by passive diffusion

**Figure 1:**
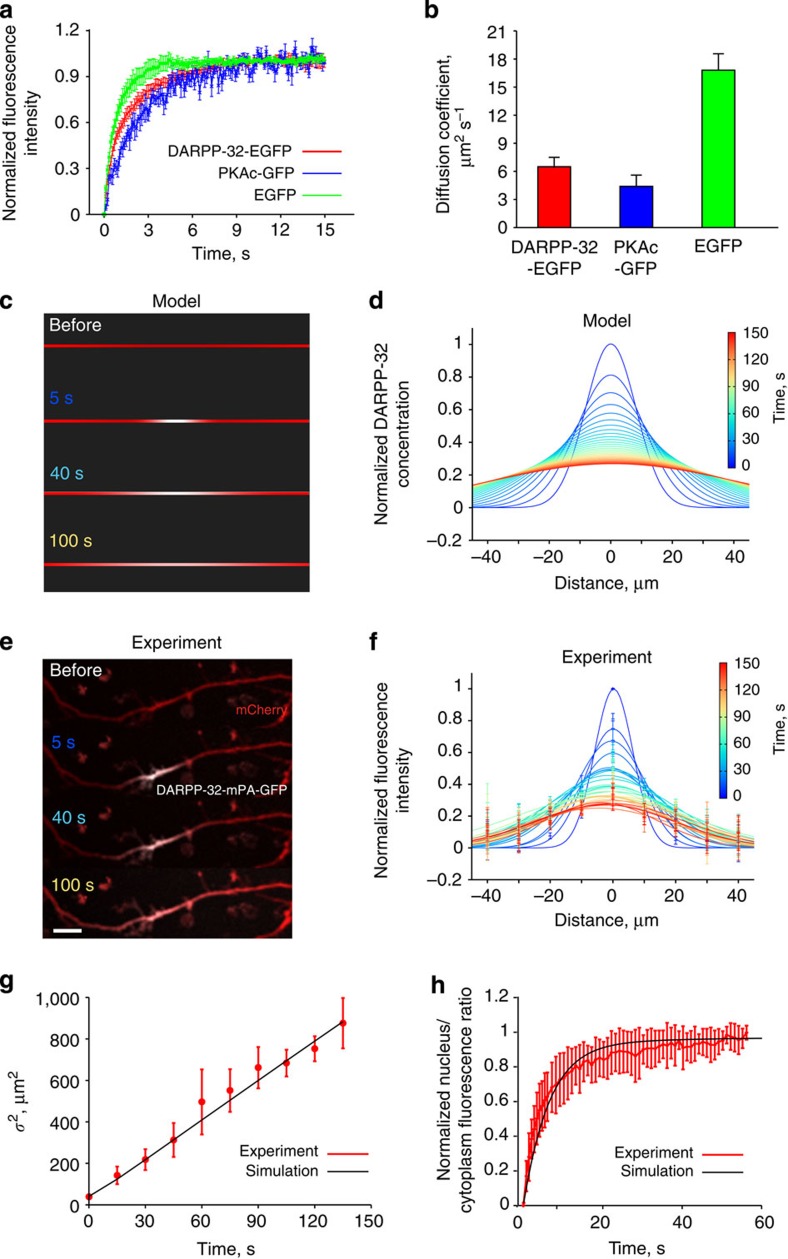
DARPP-32 moves along dendrites by passive diffusion. (**a**) FRAP, normalized to the highest fluorescence intensity (mean±s.e.m., six replicates for DARPP-32-EGFP; five for PKA-GFP; six for EGFP). (**b**) Diffusion coefficients derived from the curves shown in **a**. (**c**) Spreading of DARPP-32 molecules (white), initiated at the centre of the dendrite of a modelled neuron. (**d**) Summary of DARPP-32 fluorescence spreading and decay in the neuron modelled in **c**. (**e**) Spreading of mPA-GFP-DARPP-32 after photo-activation of the tag fluorescence in dendrites of cultured MSNs. mPA-GFP fluorescence is in white, neuronal shape is revealed by co-transfected mCherry. Scale bar, 10 μm. (**f**) Summary of DARPP-32 fluorescence spreading and decay in cultured MSNs. Data are means±s.e.m. of the average intensity for every 10-μm region (*n*=7). In **d**,**f** each colour represents a time point, with 5-s intervals, for a total duration of 150 s. (**g**) Comparison between the variance (square of sigma, μm^2^) of Gaussian curve fitting based on each time point in experiments **d** (mean±s.e.m., *n*=7) and simulation result of modelled neuron (using a diffusion coefficient of 4 μm^2^ s^−1^). (**h**) Validation of the model parameters used for DARPP-32 nuclear translocation, by the comparison of the model simulation results (black line) with our experimental observations on nuclear translocation after photoactivating cytosolic mPA-GFP-DARPP-32 fluorescence (red line, means±s.e.m., *n*=6). *R*^2^=0.93.

We first characterized DARPP-32 mobility in dendrites using fluorescence recovery after photobleaching (FRAP, Fig. 1a). The apparent diffusion coefficient of DARPP-32 tagged with enhanced green fluorescent protein (EGFP) was 6.5±1 μm^2^ s^−1^ (mean±s.e.m., *n*=6, Fig. 1b). In the same conditions we found diffusion coefficients of PKA catalytic subunit (PKAc) and EGFP to be 4.4±1.2 μm^2^ s^−1^ (*n*=5) and 16.6±2.8 μm^2^ s^−1^ (*n*=6), respectively (Fig. 1a, b), in the same range as the previously published values (Supplementary Table 1).

We next tested whether diffusion can account for DARPP-32 mobility in dendrites or whether the protein is also actively transported. To do so we compared the mobility pattern predicted from pure diffusion to experimental results. We first simulated the expected profile of tagged DARPP-32 spatial diffusion along the dendrite (Fig. 1c,d). We then expressed DARPP-32 tagged with monomeric photoactivable GFP (mPA-GFP) in cultured striatal neurons (Fig. 1e) and measured its spreading following photoactivation of a 10-μm dendrite segment. The distribution of DARPP-32-mPA-GFP at increasing times after photoactivation had symmetrical profiles, which were well fitted by Gaussian functions (Fig. 1f). The relationship between the square of the standard deviation of the Gaussian fits (*σ*^2^) and time was linear (Fig. 1g), as expected for the three-dimensional diffusion in a cylinder^25^,^26^. Activation of the cAMP pathway by a D1R agonist did not alter the shape of the fluorescence profile or speed of spreading (Supplementary Fig. 1), indicating that the activation of DARPP-32 by the cAMP/PKA pathway did not change its mobility.

We then tested whether diffusion combined with nuclear transport could account for DARPP-32 nuclear accumulation^24^. We built a spatial model taking into account active nuclear import and export of DARPP-32 and its passive diffusion through nuclear pores (see Methods). We then measured the rate of nuclear accumulation of DARPP-32-mPA-GFP after photoactivation in the perikaryon and found that it was similar to that predicted by the model (Fig. 1h). We concluded from the comparison of the various model predictions with experimental results that DARPP-32 mobility in dendrites and perikaryon results predominantly, if not only, from passive diffusion.

### Modelling predicts localized dendritic phosphoDARPP-32

**Figure 2:**
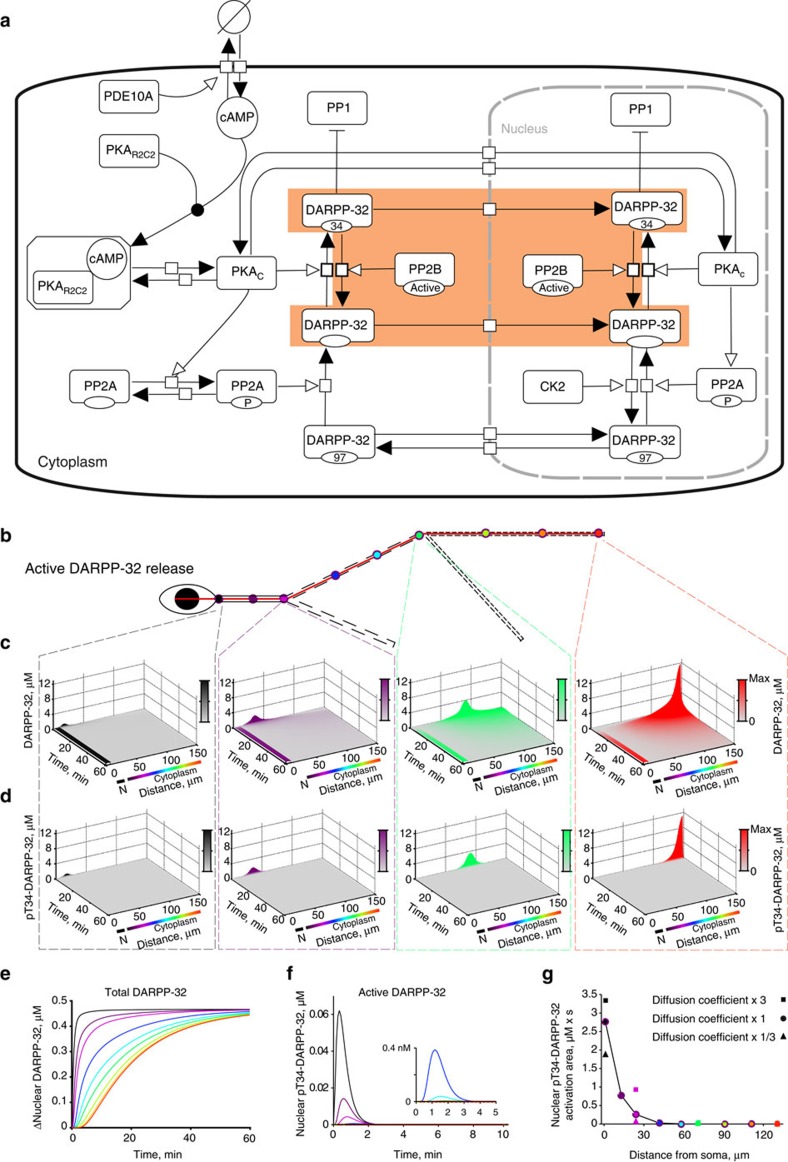
Phospho-DARPP-32 dendrites-to-nucleus reaction-diffusion model. (**a**) Model diagram of the reactions included in the model, depicted in Process description language of the systems biology graphical notation^69^. The sub-model used for the spreading of dendritically activated DARPP-32 and its dephosphorylation by calcineurin (PP2B) is highlighted in orange. (**b**) Two-dimensional representation of the modelled neuron, with the positions of the nine stimulation locations (coloured circles). The red line indicates where the spatial and temporal map of DARPP-32 was measured. (**c**,**d**) Spatial and temporal changes in the total DARPP-32 (**c**) and pT34-DARPP-32 (**d**) concentration, after pT34-DARPP-32 molecules were released at the beginning of primary (black), end of primary (magenta), end of secondary (green) and end of tertiary dendrite (red) as indicated in **b** and by dashed lines. Colour intensities are proportional to the maximum concentration (Max) reached for each simulation. N, nucleus. (**e**) Time course of the total DARPP-32 average increase in concentration (Δ) inside the nucleus, after pT34-DARPP-32 release at nine distinct dendritic locations indicated in **b**. (**f**) Same as in **e** for pT34-DARPP-32. (**g**) Integration of DARPP-32 activation, corresponding to the areas under the curves in **f**, plotted as a function of distance between the stimuli and soma. Square and triangle represent results of calculation in which the diffusion coefficient of DARPP-32 was increased or decreased threefold (that is, changing diffusion coefficient from 7 to 21 or 2.3 μm^2^ s^−1^).

We next investigated by computational modelling whether a protein, like DARPP-32, which moves by diffusion, is likely to be active in the nucleus when it is phosphorylated at Thr34 in dendrites (here the phosphorylated form is abbreviated as pT34-DARPP-32 and is referred to as the active form). We developed a reaction-diffusion model including calcineurin (PP2B), which dephosphorylates pThr34 (ref. 27) (Fig. 2a, highlighted part). This model was simulated in a three-dimensional compartment with geometrical shapes reproducing a simplified MSN dendritic arborization with realistic sizes^28^ (Fig. 2b). DARPP-32 activation was mimicked by the ‘release’ of a fixed amount of DARPP-32 molecules phosphorylated at Thr34 (pT34-DARPP-32) in a 3-μm^3^ volume at nine different dendritic locations (Fig. 2b). We calculated in each case the amount of total DARPP-32 and pT34-DARPP-32 entering the nucleus. For both, distal release led to higher local concentration and wider spreading in space and time than proximal release (Fig. 2c,d). Due to phosphatase activity, the diffusion of pT34-DARPP-32 was virtually limited to the release site and was very transient at all the dendritic locations (Fig. 2d). At the steady state (~1 h after dendritic release), the same quantity of DARPP-32 molecules had penetrated in the nucleus whatever the release location (Fig. 2e). However, only a small fraction (~1% or less) of DARPP-32 was still phosphorylated when entering the nucleus and its phosphorylation was transient (Fig. 2f). Inclusion in the model of CK2, which enhances DARPP-32 export from the nucleus by phosphorylating Ser97 (ref. 24), decreased the final amount of DARPP-32 in the nucleus but did not alter the temporal profile (Supplementary Fig. 2). In contrast, the distance between dendritic ‘release’ location and soma was the major factor determining the amplitude and duration of the wave of nuclear pT34-DARPP-32 (Fig. 2e). To summarize its overall impact on the nucleus, we used the ‘integrated activity’, that is, the concentration of the active molecule multiplied by the duration of its activation, corresponding to the area under the curve of the activation time-course^29^ (Fig. 2g). Using this presentation, it was clear that the closer to the soma the DARPP-32 molecules were released, the higher was the quantity of pT34-DARPP-32 in the nucleus. As expected, the longer the molecules travel to reach the nucleus, the higher is their probability of dephosphorylation.

These simulations were carried out using a conservative diffusion coefficient value (7 μm^2^ s^−1^) estimated from our measurements using DARPP-32-EGFP and extrapolated to untagged DARPP-32 (see Methods). To evaluate the consequences of possible imprecision in diffusion coefficient estimation, we repeated the simulation after multiplying or dividing its value by three. This procedure slightly modified nuclear pT34-DARPP-32 concentrations following proximal release but had no effect after distal release (Fig. 2g). Thus, the model was robust to changes in diffusion coefficient values. We concluded that due to phosphatase activity, the dendrite length restricts DARPP-32 phosphorylation near its activation site. Only DARPP-32 molecules phosphorylated close to the soma are predicted to enter the nucleus in their active form.

### Modelling shows nonlinear dendrite-to-nucleus signalling

**Figure 3:**
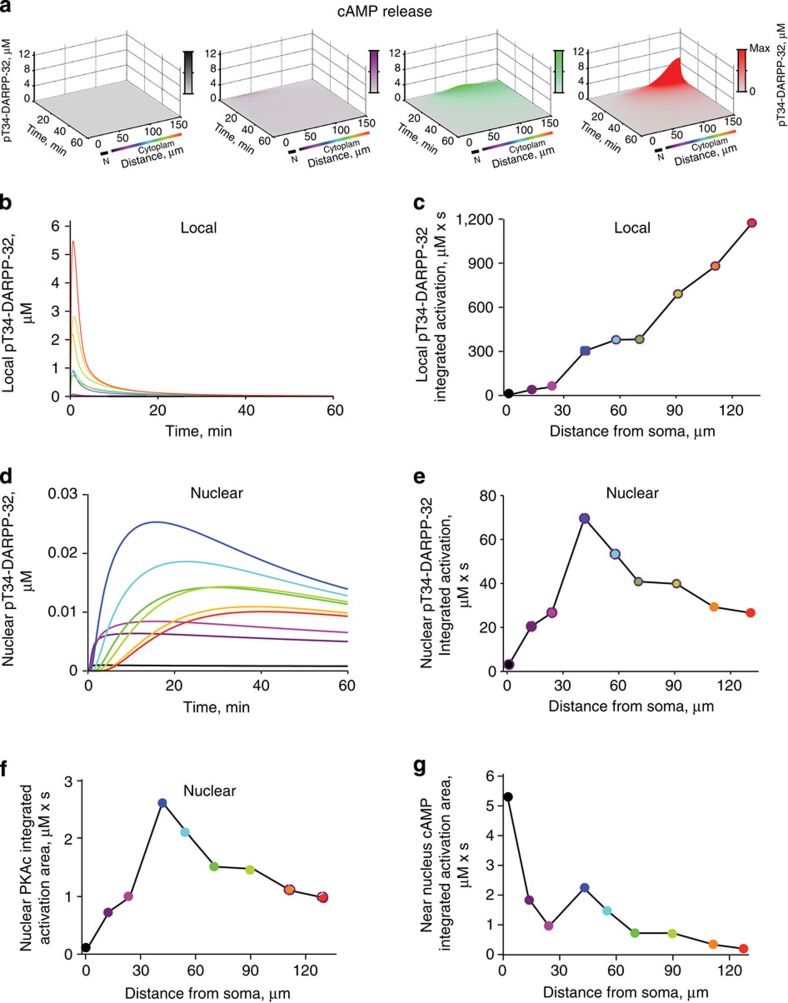
Model predicts nonlinear cAMP signalling from dendrites to nucleus. (**a**) Spatiotemporal changes in pT34-DARPP-32 concentration along the line indicated in Fig. 2b, after cAMP molecules were released in 5 s at the beginning of primary (black), end of primary (magenta), end of secondary (green) or end of tertiary dendrite (red). Colour intensities are proportional to the maximum concentration (Max) reached for each simulation. N, nucleus. (**b**) Time course of pT34-DARPP-32 concentration at the location of cAMP release. (**c**) Plot of integration of local DARPP-32 activation (area under the curve in **b**) as a function of the distance between the stimuli and soma. (**d**) Time course of pT34-DARPP-32 concentration in the nucleus. (**e**) Plot of integration of nuclear DARPP-32 activation (area under the curve in **d**) as a function of the distance between the stimuli and soma. (**f**) Integrated nuclear PKA activity (area under the curves in Supplementary Fig. 4b), as a function of the distance from the dendritic site of cAMP release. (**g**) Integrated cAMP activity (area under the curves in Supplementary Fig. 4e), as a function of the distance from the cAMP release site.

Because in our model pT34-DARPP-32 diffusing from distal dendritic sites did not accumulate in the nucleus, we tested the contribution of upstream signalling components, using a model that included all the reactions shown in Fig. 2a. Its accuracy was supported by the comparison of simulation results with published experimental observations (Supplementary Fig. 3a–d and Methods). Activation was triggered by the ‘release’ of a fixed amount of cAMP molecules at nine different dendritic locations (as in Fig. 2b). This fixed amount of cAMP mimicked the activation of D1R and subsequent stimulation of adenylyl cyclase at synapses of the same size (that is, with the same number of receptors and downstream signalling molecules) at various sites. Depending on the location, however, the model led to different patterns of pT34-DARPP-32 variations both locally and in the nucleus (Fig. 3a–e). The same amount of cAMP increased pT34-DARPP-32 locally much more efficiently in distal than in proximal dendrites (Fig. 3a,b). This was expected since following the release of the same number of cAMP molecules, the local concentration of cAMP was higher in distal dendrites due to their smaller section. Phosphorylation of DARPP-32 lasted longer and spread more (Fig. 3a) than when pT34-DARPP-32 molecules were ‘released’ in the previous model (Fig. 2d). Thus, local dendritic pT34-DARPP-32 integrated activity increased almost linearly with the distance from the soma (Fig. 3c). Intriguingly, cAMP release at various dendritic locations triggered sustained increases in nuclear pT34-DARPP-32, which did not display a monotonic relation with the distance (Fig. 3d). This was very clear when plotting nuclear integrated DARPP-32 activity as a function of the distance of cAMP release site to the soma (Fig. 3e). Nuclear DARPP-32 activity (that is, pT34-DARPP-32) did not vary in a linear uniform manner with the distance, but displayed an inverted-U shape, with a maximum for cAMP release in secondary dendrites (Fig. 3e). The model revealed that the dendritic tree influences signalling activity and leads to sharp, transient increases in local DARPP-32 activity at the stimulation point and prolonged low-amplitude nuclear responses that do not follow the order of local activities (compare Fig. 3c,e).

A major difference between the simplified (Fig. 2a, highlighted part) and complete (Fig. 2a) models is the presence of PKA in the latter, which is activated cooperatively by cAMP^30^. To examine the importance of this enzyme in shaping the effects of dendritic geometry on signalling to the nucleus, we arbitrarily increased tenfold the affinity of PKA regulatory subunit for cAMP (Supplementary Fig. 3e). This markedly altered the integrated nuclear pT34-DARPP-32 curve, which was shifted to the left, with a maximum nuclear pT34-DARPP-32 for cAMP release in the proximal dendrite (Supplementary Fig. 3f). This shows the importance of cooperative allosteric enzymatic properties for the precise shape of cytonuclear signalling. Importantly, the inverted-U shaped relationship was maintained but the peak of efficacy was shifted towards the cell body.

In the same simulations as in Fig. 3, we examined the spatiotemporal variations of PKAc concentration following cAMP release at various dendritic locations (Supplementary Fig. 4a). The plots of nuclear PKAc concentration (Supplementary Fig. 4b) and integrated PKAc activity (Fig. 3f) displayed the same variation with distance as nuclear pT34-DARPP-32 (Fig. 3d,e). To estimate the contribution of nuclear PKAc to nuclear DARPP-32 phosphorylation, we blocked PKAc nuclear translocation in the model. This decreased DARPP-32 nuclear activity by ~40% (Supplementary Fig. 4c). In combination with the results in Fig. 2e–g, this result indicated that the changes in nuclear pT34-DARPP-32 resulted from nuclear accumulation of activated PKAc for about one third and from nuclear translocation of pT34-DARPP-32 molecules activated in the vicinity of the nucleus for about two thirds.

Finally, we also plotted the spatiotemporal evolution of cAMP concentration following its release at various dendritic locations (Supplementary Fig. 4d). The increase in juxtanuclear cAMP concentration was more pronounced when dendritic cAMP release occurred near the soma and the relationship between cAMP increase and distance from the soma presented a monotonic decay (with the exception of small ‘branch effects’, see below; Supplementary Fig. 4e, Fig. 3g). These results were similar to those observed for nuclear pT34-DARPP-32 following its dendritic release in the simplified model (Fig. 2f,g).

Thus, comparison of our various modelling results suggests that when considering pT34-DARPP-32 or PKAc, the decrease in nuclear activity due to the distance between the stimulation site and the nucleus is compensated by other factors. These factors are not apparent when the stimulation is a direct ‘release’ of the active molecule measured, be it pT34-DARPP-32 or cAMP. Thus, partial compensation of distance effects appears only when multiple enzymatic reactions are included in the model, with PKA playing a critical role in the investigated pathway.

### Dendritic tree geometry shapes synapse-to-nucleus signalling

As in our full model each dendritic input consisted of the same number of cAMP molecules, it appeared that neuronal geometry contributed to the nonlinear nuclear responses. We hypothesized that different compartmental volumes resulted in nonlinear variation in chemical intermediates concentration as a function of the distance. To test this hypothesis, we simulated the effects of the same amount of cAMP released at nine locations along the main dendrite (as in Fig. 2b) of neurons of simplified shape to emphasize specific geometrical properties.

**Figure 4:**
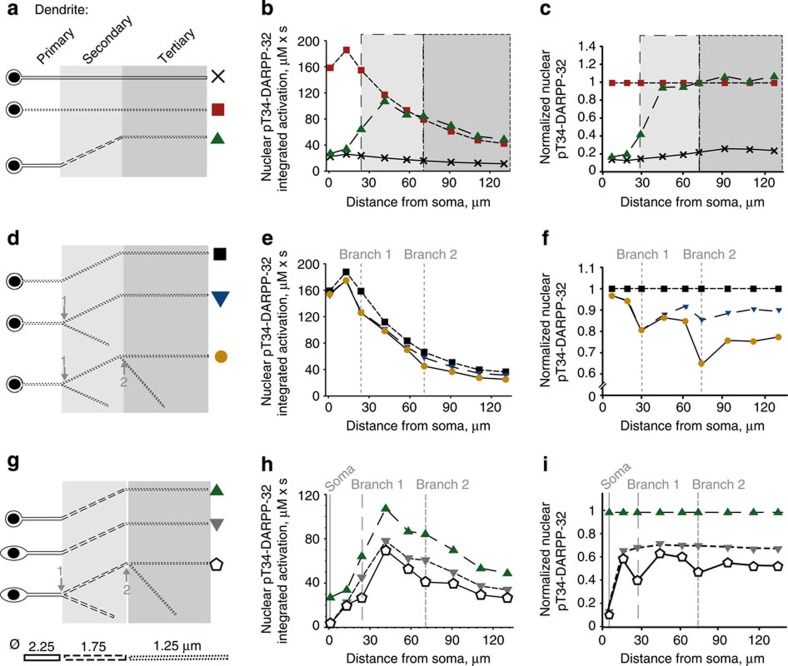
Neuronal geometry shapes dendrites to nucleus signalling. (**a**–**c**) Effects of the diameter of the dendrite. All neurons have a small soma. (**d**–**f**) Effects of branching. All neurons have a small soma and a thin dendrite (tertiary dendritic diameter). (**g**–**i**) Effects of the soma size combined with the other factors. (**a**,**d**,**g**) Geometry variations with their symbol. (**b**,**e**,**h**) Plots of the areas under the curve of the time courses of nuclear pT34-DARPP-32 concentration (as in Fig. 3e) following the release of cAMP at nine dendritic locations, as a function of the distance to the soma. (**c**,**f**,**i**) Same plot as in **b**,**e**,**h** normalized to the dendrite indicated in ordinates title. All soma and nuclei are ellipsoids. Semi-principal axes: small soma, 5.5, 5 and 5 μm; big soma: 10, 5 and 5 μm; nucleus: 4.5, 4 and 4 μm. All dendrites and branches are modelled as cylinders. Diameter of the primary dendrite (solid line): 2.25 μm; secondary dendrite (dashed line): 1.75 μm; tertiary dendrite (dotted line): 1.25 μm. Length of the dendrite at the primary location: 25 μm; secondary location: 50 μm; tertiary location: 60 μm; secondary branch location: 40 μm; tertiary branch location: 50 μm.

We first focused on dendrite diameter. We modelled a neuron with a soma 1 μm wider than the nucleus, no dendritic branch, and either a uniformly thick dendrite (primary dendrite radius) or a uniformly thin dendrite (tertiary dendrite radius) or a combination of three segments of decreasing diameter (primary, secondary and tertiary dendrite radius; Fig. 4a). For dendrites with a uniform diameter, the nuclear response was inversely related to the distance to the soma (Fig. 4b). The less efficient effect of stimulation at the closest point was due to a dilution effect in the nearby perinuclear space. The thin dendrite generated markedly higher pT34-DARPP-32 nuclear levels than the thick dendrite (Fig. 4b,c), showing that a smaller dendritic radius strengthens the stimulus effects and enhances the response in the signalling cascade. The dendrite with three segments of decreasing diameter showed a combination of the two effects: signal dilution in the large proximal segment and enhancement in the thin distal segment. This resulted in an inverted-U shape (Fig. 4b), as observed in the initial model (Fig. 3e). With the parameters used, the optimal stimulation point for triggering the highest nuclear pThr34-DARPP-32 response was located in a dendrite corresponding to a secondary dendrite.

We next examined the branching effects by modelling a neuron with a uniformly thin dendrite (radius of a tertiary dendrite) and increasing number of thin branches (Fig. 4d). Adding branches did not alter the monotonic inverse relationship between the nuclear response and the stimulus distance from the soma (Fig. 4e). A slight decrease in nuclear response was observed when cAMP was released near a branching point (Fig. 4f), accounting for indentations of the curves, as observed with the full model. We then expanded the soma size to values similar to those measured in cultured MSNs (Fig. 4g). A larger soma weakened nuclear responses, due to dilution, without changing the inverted-U shape of the nuclear activation as a function of the stimulus distance from the soma (Fig. 4h). The strongest effect was observed in the most proximal dendrite where increasing the size of the soma reduced the nuclear activity by >80% (Fig. 4i). When branching and decreasing segmental diameter were combined, the relative amplitude of the decrease due to branching was dependent on the diameter of the parent and daughters dendrites (Fig. 4i). Addition of a branch at the end of the primary dendrite decreased the nuclear impact of the stimulation by 40.7%, whereas addition of a branch to the secondary dendrite decreased the nuclear response by 32.5% (Fig. 4i). This diminution of the relative effect of branching resulted from the variation of volumes ratio as a function of the square of the dendritic radius.

**Figure 5:**
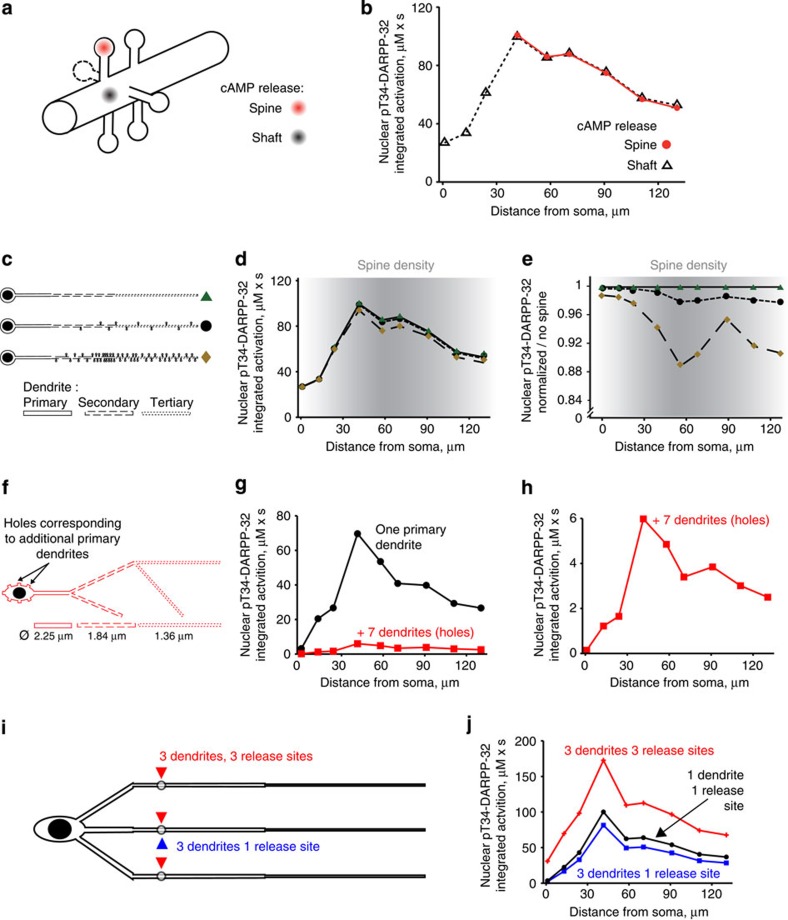
Effects of dendritic spines and arbor complexity. (**a**) Six spines at 0.5-μm intervals were added to the modelled neuron with a small soma and a straight dendrite with segments of decreasing diameter from the primary to the tertiary dendrite (as in **c** top). Spines are all identical: spherical spine head (*r*=0.5 μm) and cylindrical spine neck (*r*=0.25 μm, L=1 μm). cAMP was released in the shaft or spine heads as indicated. (**b**) Areas under the curve of nuclear pT34-DARPP-32 time course following the release of cAMP at nine dendritic locations as a function of the distance to soma. The model was not run for release sites <40 μm to soma since this region does not include spines in MSNs. (**c**–**e**) Effects of spines density on the synapse-to-nucleus signalling. (**c**) Modelled neurons with no spines (top), 10 spines (middle) or 51 spines (bottom). (**d**) Corresponding integrated activities (areas under the curve of time courses) of nuclear pT34-DARPP-32. (**e**) Same plots as in **d** normalized to the response in the absence of spine. The y axis scale is expanded to emphasize small differences. In **e** and **d** the grey shading intensity indicates spine density as reported in ref. 31. (**f**–**h**) Seven additional dendrites were modelled as truncated cylinders with the diameter of a primary dendrite, in which when molecules diffuse, they disappear (**f**, ‘holes’). Nuclear integrated activities, following cAMP release at nine dendritic locations in a neuron with one primary dendrite (as in Fig. 3e, black) or with seven additional dendrites (red) (**g**). Same red plot as in **g** with a different scale of *y* axis. (**i**,**j**) Striatal neuron modelled with three unbranched primary dendrites of decreasing segmental diameter. cAMP was released at nine different dendritic locations (as in Fig. 2b) either in one of the three dendrites (1 release site, blue) or at three identical sites in the three dendrites (3 release sites, red) (**i**). Nuclear integrated activities (**j**), following cAMP release at nine dendritic locations in a neuron with three primary dendrites. In **f**–**i**, the diameter of the secondary and tertiary dendrites was increased as compared to the initial values to mimic the presence of spines.

We also examined the effects of dendritic spines, which are distinct features of many neurons including MSNs. In our model raising cAMP in dendritic shaft or adjacent spines had similar effects (Fig. 5a,b). We then tested the consequences of adding increasing number of spines along the dendrites at densities reported for MSNs^31^. The presence of spines was equivalent to an increase in the dendrite volume, without specific alteration in the shape of the nuclear activation as a function of the distance (Fig. 5c–e). In conclusion, all tested aspects of neuronal geometry contributed to nonlinear nuclear integration of the signalling pathways activities, with a very predominant effect of decreasing dendritic diameter.

### Modelling complex dendritic trees and synaptic inputs

Our model neuron was highly simplified to remain within the computing power of VCell. We therefore specifically examined the consequences of increasing the complexity of the dendritic tree. Since D1R-expressing MSNs have on average eight primary dendrites^28^, we mimicked the presence of seven additional primary dendrites. They were modelled as truncated cylinders in which when molecules diffuse, they disappear. In this neuron the diameter of secondary and tertiary dendrites was also increased to mimic the dilution effect due to the presence of spines. This resulted in a decrease in the amplitude of the nuclear pT34-DARPP-32 response, due to a decrease in the somatic concentration of molecules, without altering the shape of the response (Fig. 5f–h). This indicated that in a real neuron with a complex dendritic tree, the effects of a single point of cAMP release, mimicking a synaptic input, have, as expected, a weaker effect than in a simpler neuron with less dendrites. Importantly this weakening did not change the non-monotonic, inverted-U shaped relationship between the distance of stimulation and the nuclear response.

It has been estimated that each striatal MSN receives on average 954 dopamine synapses^32^ originating from 95–194 dopamine neurons^33^. This indicates that each dopamine neuron provides 5–10 synapses to the same MSN. We therefore evaluated whether the stimulation of several inputs could compensate the dilution effect of a complex dendritic tree. We modelled a MSN with three simplified primary dendrites (Fig. 5i). Increasing the number of dendrites from one to three decreased the nuclear pT34-DARPP-32 response to cAMP release at a single site. However, stimulating the three dendrites led to a stronger nuclear response through an additive effect on concentrations in the cytoplasm (Fig. 5j). Importantly these various modifications did not alter the inverted-U shape of the nuclear response. These simulations show that, as expected, increasing the complexity of the dendritic tree decreases the relative weight of single synaptic inputs through dilution effects. However, this attenuation does not alter the non-monotonic relationship between the distance of stimulus from the nucleus and the nuclear response, and can be compensated by the stimulation of several synapses.

### Experimental dendritic stimulation effects on nuclear PKA

Our modelling results predicted a nonlinear, non-monotonic relationship between the nuclear signalling and the distance from the soma. To test this prediction, we used striatal neurons in primary culture and high-resolution imaging and photolysis that enable spatial control and measurement of second messengers and protein kinase activity. We first verified that the morphological characteristics of the dendrites of the neurons corresponded to those of the model neuron (Supplementary Table 2). We also tested the influence of the variation in dendrite diameter on the model by running the simulation (as in Fig. 3d,e) for ‘extreme’ neurons, which combined all the maximal (big neuron) or minimal (small neuron) values observed in our culture conditions (Supplementary Fig. 5). As expected from the previous results showing the importance of concentration, increasing the volume decreased the amplitude of the nuclear response, whereas decreasing the volume had the opposite effect. Importantly the inverted-U shape of the relationship between the distance of the stimulus and the nuclear response was observed in both the cases. Interestingly, the site of maximal relative efficacy of cAMP to activate the nuclear response was shifted away from the cell body by increasing the volume (Supplementary Fig. 5).

**Figure 6:**
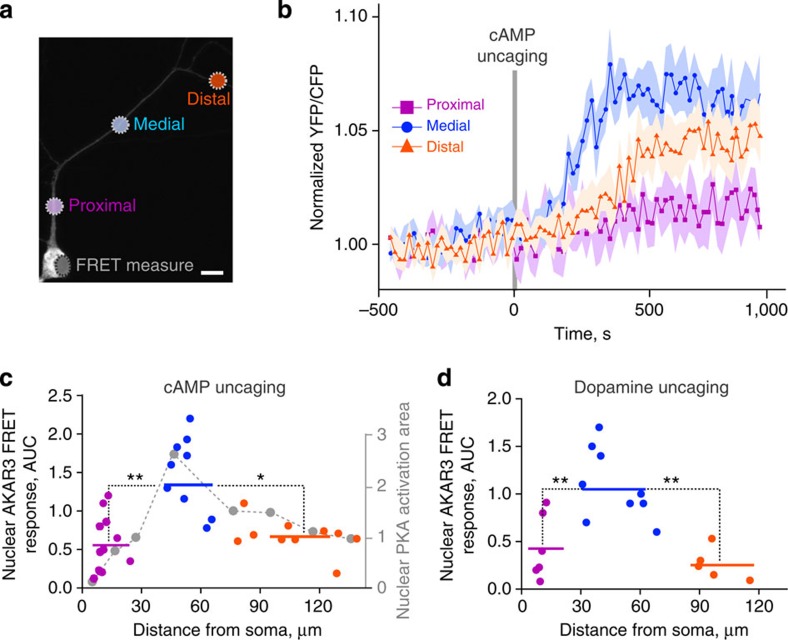
Effects of dendritic cAMP or dopamine release on nuclear PKA. Live imaging of nuclear PKA activity shows a nonlinear, non-monotonic relationship with the distance of dendritic cAMP or dopamine release from the cell body. (**a**) Striatal neurons in culture were infected with Sindbis-AKAR3 and 6 h later loaded with DMNB-caged-cAMP. cAMP was released with a brief (1 s) ultraviolet pulse (*d*=1 μm) at the indicated locations. Two stimulations at different locations (randomized order between proximal, medial and distal locations) were carried out per neuron, with an interstimulus interval of at least 30 min. Scale bar, 10 μm. (**b**) Time course of normalized 480 nm/535 nm fluorescence ratio of nuclear AKAR3, following the local release of cAMP at various locations in dendrites. Data are means±s.e.m., *n*=9–12 in four different experiments. (**c**) The experimental points (area under the curve (AUC) in **b**, colour) are plotted with the model prediction (Fig. 3f, grey dashed line; the correspondence of ordinate values is arbitrary). Comparison of the experimental points grouped as indicated by the colour code (horizontal bars correspond to means: proximal, 12±2 μm from the nucleus, mean±s.e.m., *n*=12, medial, 53±3 μm, *n*=9, and distal 108±7 μm, *n*=10), one-way analysis of variance (ANOVA), *F*_(2,28)_=5.89, *P*=0.007; Tukey’s test, *P<0.05, **P<0.01. (**d**) Striatal neurons transfected with D1R receptor, expressing AKAR3 as in **a**, were incubated with 100 μM ((N)-1-(2-nitrophenyl)ethylcarboxy-3,4-dihydroxyphenethylamine-caged-dopamine, which was photo-released as in **a** at the indicated distances from the nucleus. Comparison of the experimental points grouped as proximal, medial and distal, as indicated by the colour code (horizontal bars correspond to means: proximal, 10.3±0.7 μm, *n*=6, medial, 48.9±4.9 μm, *n*=9, distal, 101.6±4.9 μm, *n*=5, from 3 different experiments). FRET responses: 1-way ANOVA, *F*_(2,17)_=11.40, *P*=0.0007; Tukey’s test, **P<0.01.

Since our model showed the same nonlinear relationship between the nuclear PKA activity and the distance from cAMP release location to the nucleus as for pThr34-DARPP-32 (Fig. 5e), we measured the PKA activity with AKAR3 biosensor coded by a Sindbis viral vector^34^. Cultured striatal neurons were incubated for 20 min with cell-permeant caged cAMP (4,5-dimethoxy-2-nitrobenzyl adenosine 3′,5′-cyclicmonophosphate, DMNB-cAMP), and the localized intracellular cAMP release was triggered by a ultraviolet laser light pulse. Nuclear PKA activity was measured by confocal fluorescence resonance energy transfer (FRET) imaging. Global uncaging in the whole neuron by a 1-s ultraviolet light pulse produced a transient AKAR3 phosphorylation with an amplitude comparable to that induced by bath application of the adenylyl cyclase activator forskolin, thus validating the technique (Supplementary Fig. 6a,b). We then uncaged cAMP locally in a restricted area, at three different dendritic locations: proximal, medial, and distal, roughly corresponding to primary, secondary and tertiary dendrites (Fig. 6a). cAMP release in the medial segment produced the strongest AKAR3 phosphorylation in the nucleus (Fig. 6b,c). Nuclear PKA activity displayed different kinetics depending on the release site, with slower signals of reduced amplitude when cAMP was uncaged in distal dendrites as compared with medial dendrites (Fig. 6b). After cAMP photolysis, we verified the ability of neurons used for the three types of stimulation to similarly activate nuclear PKA, by measuring their response to bath-applied forskolin (Supplementary Fig. 6c). Moreover, we confirmed that the FRET response was due to the phosphorylation of the probe, by verifying that no nuclear activity was detected after cAMP release at the three dendritic locations in the presence of a PKA inhibitor, H89 (Supplementary Fig. 6d). Thus, experimental results confirmed the predictions of the model, showing the strongest activation of nuclear PKA by cAMP release in secondary-like dendrites.

We then tested whether the stimulation of membrane receptors could give the same pattern of signalling responses as cAMP uncaging. Striatal neurons in cultures expressing AKAR3 were transfected with D1R to ensure a homogenous expression of the receptor and incubated with NPEC-caged-dopamine ((N)-1-(2-nitrophenyl)ethylcarboxy-3,4-dihydroxyphenethylamine, 100 μM). Dopamine was released by a 1-s ultraviolet light pulse at various locations with respect to the dendritic tree and AKAR3 FRET measured in the nucleus (Fig. 6d). Dopamine release in medial (secondary like) dendrites was more efficient to increase nuclear AKAR3 phosphorylation than in proximal or distal dendrites, confirming and extending the results with cAMP release.

**Figure 7:**
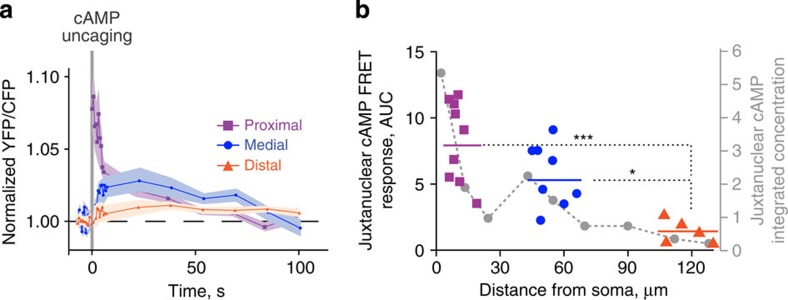
Effects of dendritic cAMP release on juxtanuclear cAMP. Juxtanuclear cAMP concentration is monotonically, inversely related to the distance between soma and the site of dendritic cAMP release. Striatal neurons in culture were infected with Sindbis-TEPACVV and 6 h later loaded with DMNB-cAMP (caged cAMP). cAMP was uncaged with a brief (1 s) UV pulse (*d*=1 μm) in proximal, medial or distal dendrites (as in Fig. 6a). (**a**) Time course of normalized 480 nm/535 nm fluorescence ratio of juxtanuclear TEPACVV, following local release of cAMP at various locations in dendrites. (**b**) Comparison of the experimental points (area under the curve in a) and the model prediction (from Fig. 3g, grey dots and dashed line). Data are grouped as proximal, medial and distal, as indicated by the colour code (horizontal bars correspond to means: proximal, 9.9±1.3 μm, *n*=9, medial, 53.3±2.5 μm, *n*=8, distal, 116.4±4.3 μm, *n*=5, from three different experiments). One-way analysis of variance , *F*_(2,19)_=12.91, *P*=0.0003; Tukey’s test, *P<0.05, ****P<10^−4^.

Since our modelling data predicted that the juxtanuclear cAMP concentrations should vary monotonically inversely with the distance from the soma, we tested this prediction experimentally, using TEPACVV, a biosensor whose FRET varies with cAMP binding^35^. In this experiment, the FRET signal decreased with the distance between soma and the site of cAMP uncaging (Fig. 7a,b). These combined experimental results confirmed the existence of a complex nonlinear, non-monotonic relationship between the cell body/nucleus response and the distance of the stimuli on the dendrites for PKA-mediated phosphorylation, but not for cAMP levels.

### Dendritic cAMP release effects on histone 3 phosphorylation

**Figure 8:**
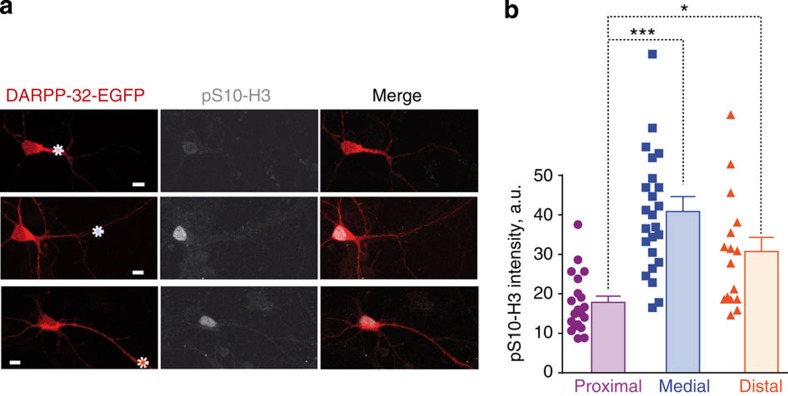
Effects of dendritic cAMP on histone 3 phosphorylation. H3 phosphorylation shows a nonlinear relationship with the distance of dendritic cAMP release from the cell body. (**a**) Striatal neurons in culture transfected with DARPP-32-EGFP, loaded with DMNB-cAMP and treated as in Fig. 6a, were fixed at the end of the experiment after precise localization of their position on the coverslip. On the examples shown the site of ultraviolet cAMP uncaging is indicated by a dotted circle. Histone H3 Ser10 phosphorylation was detected by immunofluorescence with phospho-specific antibodies (pS10-H3). (**b**) pS10-H3 immunofluorescence was quantified for stimulation sites corresponding to proximal, medial or distal dendrites. Individual data points are shown, as well as means+s.e.m. One-way analysis of variance, *F*_(2,59)_=3.74, *P*<0.03; Tukey’s test, *P<0.05, ***P<0.001.

To test the potential functional impact of the relationship between the distance of stimulus and the nuclear signalling responses, we examined the efficiency of cAMP released at various locations to induce phosphorylation of an endogenous nuclear substrate. We chose histone 3 (H3), a resident nuclear protein whose phosphorylation on Ser10 depends on the cAMP/PKA/DARPP-32 pathway in MSNs^24^. Cultured striatal neurons were loaded with DMNB-cAMP and stimulated with a 1-s ultraviolet light pulse at a single dendritic location, different from one neuron to the other (Fig. 8a). Fifteen minutes later neurons were fixed, their position was identified and pS10-H3 was measured by immunofluorescence with phospho-specific antibodies. cAMP uncaging at medial and distal dendrites locations produced a higher-intensity pS10-H3 immunoreactivity than that produced by the stimulation at proximal positions (Fig. 8a,b). Thus, the study of an endogenous substrate of the signalling pathway also disclosed a nonlinear, inverted-U shape relationship of nuclear signalling response with the distance of the stimulus location in dendrites.

## Discussion

Our study combined computer modelling and live imaging of striatal neurons to examine nuclear responses triggered by the activation of the cAMP pathway at various distances from the soma in dendrites. We show that, unexpectedly, the efficacy of cAMP production to trigger nuclear responses does not simply decay with the distance from the soma, but is markedly nonlinear because of dendrites geometry. We identify biochemical and morphological parameters that produce this nonlinearity and determine simple principles likely to have general applicability. Formally our results emphasize some similarities between the complex biochemical pathways in dendrites and aspects of linear cable theory^7^,^8^, which should be an interesting area for further studies.

Communication from synapses to the nucleus participates in a broad range of processes including the regulation of spine dynamics, modulation of dendritic complexity and regulation of long-term changes in synaptic strength, which all require transient and/or long-lasting changes in transcription^4^,^5^,^6^. Active transport achieves a rapid transfer of some specific signalling molecules over long distances, usually in multiprotein complexes that can prevent their inactivation^36^,^37^. In contrast, it has been suggested that passively diffusing, labile signalling molecules can only function in the vicinity of their site of generation and that successful signal propagation to the nucleus depends on the distance between the nucleus and the site of activation^38^. We studied a well-characterized cAMP-activated pathway involving cAMP, PKA and DARPP-32, a PP1 inhibitor that plays a key role in dopamine-innervated neurons of the striatum^16^,^17^. This pathway regulates many cytoplasmic responses and can reach the nucleus^24^. Our experimental results showed that DARPP-32 displacement can be accounted for by passive diffusion, providing an excellent framework to understand the properties of passively diffusing signalling molecules in neurons. Locally generated diffusible molecules are diluted in the cell volume and their concentration in the nucleus depends on its distance from their site of production. Moreover, if the active form of the molecule is unstable, like pT34-DARPP-32 that is readily dephosphorylated^39^,^40^, its ability to reach the nucleus is strongly limited by the distance travelled. Our simulations based on experimentally validated parameters show that pT34-DARPP-32 does not reach the nucleus in significant amounts when it is generated >30 μm away from the soma. In contrast, we find that signals can be efficiently transferred to the nucleus by the diffusion of cAMP and PKA. The cAMP pathway can reach the nucleus^41^,^42^ but spreading of cAMP signalling is limited by phosphodiesterases^9^,^43^,^44^ and binding to target sites^45^. Such regulators are expected to be major sources of distance-dependent attenuation of signalling pathways propagation in dendrites. Accordingly, our model and our experiments show that cAMP concentration in the cell body is roughly inversely related with the distance of its site of generation.

In contrast, our modelling study revealed that dendritic tree morphology has the potential to amplify signals downstream from cAMP and to counterbalance the distance effects. Our numerical modelling shows that the main effects of dendritic shape arise from the volume differences between the dendritic segments. Thin dendrites amplify biochemical signals because the same amount of second messenger molecules induces larger concentration changes that are more efficient for pathway activation, in agreement with studies on the general effects of cell shape on signalling^9^,^10^. The soma and large primary dendrites, in contrast, act as a sink where cAMP is rapidly diluted and may not reach the PKA activation threshold. Increasing the affinity of PKA for cAMP in the model shifted the optimal site of stimulation towards the cell body, showing the importance of this parameter in shaping synapse-to-nucleus signalling. Similar reasoning may apply to other concentration-dependent enzymatic reactions. This underlines the potential role of scaffolding proteins, such as PKA-anchoring proteins, expected to prevent dilution effects by local enrichment of relevant proteins^22^. We found that dendritic branching has much less effects than diameter, through local dilution. As in the case of impedance mismatch for electrical potential^46^,^47^, the relative effects of branching are stronger for larger branches since its importance increases with the ratio of the square of the diameters. In general, for the same number of cAMP molecules generated, the local efficiency of signalling pathways activation is inversely proportional to the diameter of the dendrite. This may have significant consequences as strong cAMP signalling is important for spine formation^48^. It may also contribute to the phosphorylation of different ion channels in proximal and distal dendrites and their functional consequences^49^,^50^,^51^.

A major unexpected finding in our work is that when diffusible labile messengers are generated in dendrites their effect on the nucleus (and perikaryon) does not decay monotonically with the distance. Instead, the opposing influences of distance-induced attenuation and increasing local activation with decreasing dendrite diameter result in an asymmetrical inverted-U shape curve. There is an optimal location for a synaptic input to influence nuclear responses. The precise location of this optimum is likely to vary from one neuronal type to the other according to the specific values of morphological and biochemical parameters. Mimicking the complex dendritic tree of an MSN attenuated the relative influence of a single synaptic input on nuclear responses without changing its U shape relationship with distance. However we provide evidence that this attenuation is likely to be compensated in physiological situations by the simultaneous activation of multiple synaptic DA inputs^32^,^33^. Using experimentally determined parameters for striatal MSNs we found an excellent correspondence between our model predictions and the responses we observed by live imaging. cAMP released in medial (secondary-like) dendrites was much more efficient to increase nuclear PKA activity, than in proximal or distal dendrites. Similar results were also observed when the stimulus was locally uncaged extracellular DA acting on D1R. The stronger effects of cAMP in secondary-like dendrites, as compared with primary- or tertiary-like dendrites, was also apparent when measuring phosphorylation of histone H3, an endogenous nuclear target of the pathway. This latter observation stresses the functional consequences of signalling responses modulation by the shape of the dendritic tree.

Combined biochemical and geometrical parameters create a complex integration pattern of signalling in dendrites that counterbalances the passive filtering properties of dendritic length. Our results reveal that the geometry of the dendritic tree makes an additional and unexpected contribution to the computational power of neurons in terms of signalling from synapses to the soma and nucleus. We used as a model in our study a well-characterized signalling pathway in striatal projection neurons. Since similar biochemical and morphological features are shared by virtually all neurons, it is likely that our results have general implications for diffusion-mediated signalling pathways. Specific values of biochemical and geometric parameters in each neuron will condition its particular synapse-to-nucleus integration properties. Our results open novel prospects in the study of signalling from synapses to nucleus by revealing how molecular signals can be shaped by basic geometrical properties of dendrites.

## Methods

### Primary striatal culture, transfection and reagents

Striatal neurons from 14.5-embryonic day Swiss mice (Charles River Laboratories) were placed in 35-mm diameter wells and grown in Neurobasal medium (Gibco 21103-049) supplemented with B27 (1x, Gibco 17504-044), GlutaMAX (500 μM, Gibco 35050-061), penicillin (50 IU per ml) and streptomycin (50 μg ml^−1^) at 37 °C in 5% CO_2_. After 7–10 days in culture, striatal neurons were transfected using Lipofectamine LTX with Plus Reagent (Invitrogen A12621) in Neurobasal medium without supplements. All treatments or imaging experiments were performed 24 h later in ‘imaging medium’: MEM (Gibco 51200-046) with sodium bicarbonate (4 mM, Sigma S5761), HEPES (20 mM, Gibco 15630-056), GlutaMAX (2 mM), D-glucose (33 mM, Sigma G8270), B27 and N-2 (1x, Gibco 17502-048).

### FRAP and photoactivation experiments

Cultured striatal neurons were transfected with a plasmid coding for DARPP-32 fused with carboxy terminal of EGFP (2.5 μg per 35-mm diameter dish) or PKA-GFP (2.5 μg) or EGFP (2.5 μg). FRAP and all imaging experiments were carried out at 37 °C at least 24 h later on a confocal SP5 II upright microscope (Leica Microsystems) with a 40X HCX APO (0.80 NA) water objective at the *Institut du Fer à Moulin* Cell and Tissue Imaging facility. FRAP and photoactivation experiments were performed with the FRAP Wizard software (Leica Microsystems). During acquisition, the temperature of the sample and the objective was maintained at 37 °C in a heated chamber. The pinhole aperture was opened in such a way that the full width at half the maximal intensity was larger than the depth of the cell. Before photobleaching, 30 images were taken at 0.116-s intervals. After photobleaching of a circular region for 500 ms, 500 images were taken at 0.116-s intervals.

### FRAP data analysis and calculation of diffusion coefficients

In experiments, a circular region within the cytoplasm was photobleached. The average fluorescence density of the bleached region in each image was corrected by deducing the average fluorescence density of the background taken at the same time. The diffusion coefficients were calculated by using a published method^52^, taking into account the radius of the bleached area, the half recovery time and a correction constant calculated based on the laser intensity. The bleached area was analysed on the first post-bleaching image. The radius (*ω*) of the bleached area was measured within a region where fluorescent intensity was <1e^−2^ of the intensity before bleaching and this was measured for each individual experiment. On average, the bleaching radius was 3 μm for DARPP-32-EGFP, 3 μm for PKA-GFP and 4.4 μm for EGFP. The half recovery time (*τ*_d_) was obtained by fitting the fluorescence intensity during recovery into the equations proposed by Feder *et al*.^53^ (equation (1); see Supplementary information for reference). Fixed samples were also bleached, to calculate the kinetics of the bleaching, based on the fluorescence intensity before and after bleaching (but without diffusion during bleaching period) and the assumption that bleaching is a first-order chemical reaction. This procedure enabled us to estimate the correction constant (*γ*) based on the relationship between *γ* and the kinetics of bleaching drawn by Axelrod *et al*.^52^. Finally, diffusion coefficients were determined based on equation (2).


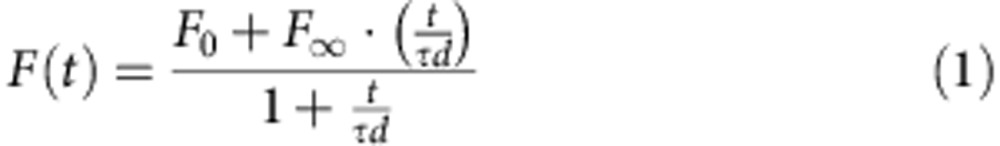



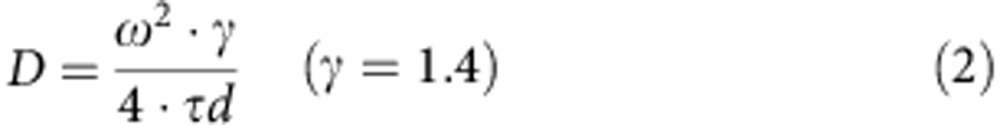


### Diffusion and model of photoactivable-GFP-DARPP-32

Cultured striatal neurons were co-transfected with mCherry (1 μg DNA) and DARPP-32 fused to mPA-GFP (1.5 μg DNA). A day after transfection, mPA-GFP fluorescence was activated by two 100-ms ultraviolet light exposures at 0.663-s interval, in a 10-μm long rectangular region at the centre of the dendrite (in total 90 μm long, no branches, with unified diameter) in mCherry-positive cells. For SKF81297 (Sigma-Aldrich) treatment, cells were co-transfected with mCherry (1 μg DNA), DARPP-32-mPA-GFP (1.5 μg DNA) and D1R (0.5 μg) DNA. Ultraviolet activation was carried out 15 min after SKF81297 (10 μM) application. Post-activation pictures were taken at 5-s intervals for a duration of 150 s. The average fluorescence intensity of every 10-μm side rectangular dendritic region was measured at all time points; after background fluorescence subtraction, the intensity was normalized to the first post-photoactivation image. A computational model restricted to DARPP-32 diffusion was developed, incorporating a simple geometry (ellipsoid soma with a cylindrical dendrite, diameter (*d*), 1.75 μm, length (*L*), 180 μm). DARPP-32 (50 μM) was released in a 10-μm-long region 70 μm from the soma. Simulation results were quantified in the same way as the experiments.

For nuclear translocation experiments, cultured striatal neurons were co-transfected with mCherry and DARPP-32-mPA-GFP, as described above. Ultraviolet light was used (during 100 ms) at multiple points in the soma. Ten pre-activation and 10 post-activation pictures were taken with 0.663-s intervals, followed by 50 frames with 1-s intervals. The ratios between nuclear and cytoplasmic average fluorescence intensity were calculated (after background fluorescence subtraction at every time point). The ratios were normalized between the minimum and maximum ratios obtained for each experiment. A computational model was set up combining DARPP-32 diffusion and cytonuclear translocation via passive diffusion and active transportation. Simulation was initiated with 50 μM pS97-DARPP-32 in the soma (within the standard geometry, Fig. 2b). Simulation results were analysed similarly as the experimental data.

The diffusion constant of DARPP-32 used in modelling was based on our experiments. The diffusion coefficients of other proteins and protein complexes were based on published values or estimated by their molecular weights relative to DARPP-32, by assuming that the ratio of diffusion coefficients is in reverse relationship with the protein Stokes radius *S*_R_ (Supplementary Table 1). The *S*_R_ values used were 3.4 nm for DARPP-32 and 2.8 nm for GFP. If these values were additive the expected diffusion coefficient for the untagged DARPP-32 would be 12 μm^2^ s^−1^. However, DARPP-32 is an unfolded protein^54^,^55^ with a presumably lower diffusion than would be predicted for a globular protein. Since we found diffusion coefficients of DARPP-32-GFP of 6.5 and 4 μm^2^ s^−1^ by FRAP and photoactivation, respectively, we used a conservative value of 7 μm^2^ s^−1^ for untagged GFP in our model, unless otherwise indicated.

### FRET and local photolysis

Striatal neurons were infected, after 7–10 days in culture, with Sindbis virus encoding EKAR3 or T-EPAC-VV (mTurquoiseΔ-Epac(CD,ΔDEP)-^cp173^Venus-Venus) 6–8 h before the imaging experiments. For cAMP photolysis, 20 min before the experiment culture medium was replaced by ‘imaging medium’. DNMB-cAMP (50 μM) or NPEC-dopamine (100 μM) was added to the imaging medium for cAMP or dopamine photolysis. FRET experiments were carried out at 37 °C on a confocal SP5 II upright microscope (Leica Microsystems) with a 40X HCX APO (0.80 NA) water objective, at the *Institut du Fer à Moulin* Cell and Tissue Imaging facility. FRET biosensors were excited at 456 nm and fluorescence measured simultaneously in cyan fluorescent protein (CFP, 460–515 nm) and yellow fluorescent protein (YFP, 525–600 nm) channels. cAMP was uncaged at a single 1-μm diameter point with a ultraviolet laser (405 nm) for 1 s. Images were analysed with custom software in Matlab (Matworks). In regions of interest the emission ratio was calculated for each pixel (YFP/CFP for AKAR3 and CFP/YFP for T-Epac-VV) and coded in hue in pseudocolour images.

### Computational modelling

*Model structure and validation*. PKA activation was modelled as a sequential binding of four cAMP molecules to PKA whole enzyme, followed by the release of two active catalytic subunits. The parameters for these processes were validated against published experimental data^30^. PDE10A was the only phosphodiesterase considered in the model, as it is the predominant isoform in D1R-expressing MSNs^56^. The parameters concerning its hydrolysis ability were validated against the published values^57^. Two phosphorylation sites of DARPP-32 were considered in the model: Thr34 phosphorylated by PKA and Ser97 phosphorylated by CK2. Thr34-phospho-DARPP-32 competitively inhibits PP1 (ref. 58). This site is dephosphorylated by calcineurin^27^. Basal phosphatase activity of calcineurin was included. Ser97 was predominantly phosphorylated at the basal state, resulting in 1.7-fold higher concentration of DARPP-32 in the cytoplasm than in the nucleus. This ratio was based on our own immunofluorescence measurements. Ser97 is dephosphorylated by PP2A (refs 24, 59), a phosphatase activated by PKA^60^,^61^. The parameters applied to the phosphorylation and dephosphorylation of both Thr34 and Ser97 were verified against published experimental data^24^.

CK2 was distributed only inside the nucleus of striatal neurons^24^, unless otherwise indicated. CK2 phosphorylates DARPP-32, enabling its active nuclear export^24^. The active nuclear import and export of DARPP-32 were modelled as two first-order chemical reactions, a simplified method which does not consider saturation, used in similar models^62^,^63^. Passive diffusion-induced nuclear translocation of DARPP-32 was also modelled as first-order reactions, since DARPP-32 is a small molecule of 23 kDa (UniProt: Q9UD71), with a Stokes radius of 3.4 nm, smaller than the estimated radius of nuclear pore complex (4.4–6.1 nm). DARPP-32 nuclear translocation rates were validated against our own experiments. Protein and protein complexes whose molecular weights are >60 kDa were not considered to travel through the nuclear pore passively. On the basis of this assumption, besides DARPP-32, only the PKAc undergoes passive diffusion-mediated nuclear translocation in the model, as indicated previously^64^. Two first-order chemical reactions were used to model PKA translocations with parameters estimated by an approach similar to the one used for DARPP-32 and were validated with the published data^65^. In the model, all the proteins in the system, including the PKAc, could undergo active transportation when they formed protein complexes with DARPP-32.

*Modelling and simulation software*. A three-dimensional reaction-diffusion model was set up in the Virtual Cell Modelling and Simulation Framework^66^. Analytical geometries were defined according to measurements from electron microscopy studies^28^,^31^ and direct measures of cultured MSN. The modelled neuron was composed of a soma (an ellipsoid with semi-principal axes of 10, 5 and 5 μm), a nucleus (an ellipsoid with semi-principal axes of 4.5, 4 and 4 μm) and a single dendritic tree. Unless otherwise specified, there were three sections on the dendrite and two branches, all were modelled as cylinders: they were primary (*d*=2.25 μm; *L*=25 μm), secondary (*d*=1.75 μm, *L*=50 μm), tertiary dendrite (*d*=1.25 μm, *L*=60 μm), secondary branch (*d*=1.75 μm, *L*=40 μm) and tertiary branch (*d*=1.25 μm, *L*=50 μm). The angles between the different principle dendritic sections were 160°. Both the branches formed 45° angles with the principle dendrites.

In total, there were 59 chemical entities and 126 reactions that were included in the model. Reactions were modelled as mass action law processes. Each enzymatic reaction was represented by the three elementary steps of binding, dissociation and catalysis. Values are listed in Supplementary Tables for initial concentrations and diffusion coefficients (Supplementary Table 3 and 4), kinetic constants (Supplementary Table 5) and nuclear translocation rates (Supplementary Table 6). The model was simulated spatially with a 0.5-μm^3^ mesh. cAMP ‘release’ signal was implemented by converting species ‘cAMPsource’ into cAMP via a first-order chemical reaction. This ‘cAMPsource’ corresponded to caged DMNB-cAMP and the chemical reaction mimicked ultraviolet light-induced uncaging. To release the same amount of cAMP in different simulations, the same concentration of ‘cAMPsource’ was placed in the same number (24) of cubic meshes for 5 s. A fully implicit finite volume, regular grid (variable time step) method^66^ was used in all simulations. The core of the model can be found in the Virtual Cell public database via VCell software under user luli with model name: Li2015_geometryCreatesNonlinearNuclearPKA.

*Computational model validation*. To validate the parameters involved in PKA activation, a compartmental model that contains only PKA activation and its activity, was set up in COPASI^67^. To start the simulation, 10 nM PKA whole enzyme were mixed with 200 μM kemptide and cAMP with varying concentrations. The enzyme activity was calculated as the concentration of phospho-kemptide after a 2-min simulation in agreement with experimental procedures^30^. Additional parameters, used to enable this fitting, were the binding, dissociation of PKA to kemptide and the catalytic constant of PKA phosphorylates kemptide (Supplementary Table 1). The simulation results were in agreement with the experimental observations (Supplementary Fig. 3a).

The catalytic ability of PDE10A was tested in a compartmental model, which initially contained 20 μM cAMP and 40 nM EPAC2. Only the high-affinity binding site of EPAC2 was included, because in the experiment that we aimed to compare with, Nikolaev *et al*.^57^ fused a single binding domain of EPAC2 in between CFP and YFP for generating a FRET signal. The affinity between EPAC2 and cAMP used in the model was 1.2 μM. After the binding of cAMP to EPAC2 reached equilibrium, 10 μM of PDE10A was added, causing the dissociation between EPAC2 and cAMP. The dissociation curve was compared with the experimental data based on the same conditions^57^. The half-time of the dissociation between EPAC2 and cAMP, according to the simulation, was ~3 s, in agreement with experimental observations (Supplementary Fig. 3b, Supplementary Table 1).

The time course of DARPP-32 phosphorylation on Thr34 and dephosphorylation on Ser97 were validated by simulating the whole spatial model with persistent elevation of cAMP concentration in the cytosol, to mimic the effect of forskolin bath application observed experimentally^24^ (Supplementary Fig. 3d). The basal phosphatase activity of calcineurin was assessed by applying a transient cAMP increase to the cytosol region for 5 min, followed by an exponential cAMP decay. The dephosphorylation of DARPP-32 at Thr34 is similar to the time course obtained experimentally after SKF application^24^.

### Estimation of nuclear translocation rates

The rate of DARPP-32 actively imported into the nucleus was estimated based on: the active transportation rate of GFP fused with nuclear localization signals (90 molecules per nuclear pore per second) and the assumption that there were ~2,000 nuclear pores per nucleus (Supplementary Table 1). This rate was then adjusted by estimating the fraction of DARPP-32 that can bind to importin alpha, given the concentration of importin alpha (~1 μM) and the affinity between importin alpha and its substrate (Kd=16 μM). The speed of passive diffusion-induced DARPP-32 going in or out of the nucleus was calculated based on the formula proposed by Renkin^68^ (equation (3)), which takes into account the Stokes radius of DARPP-32 (*r*_x_=3.4 nm), its diffusion coefficient (*D*_x_=7 μm^2^ s^−1^), the average radius of a nuclear pore (*r*_p_=5.33 nm), the nuclear pore area (*A*_p_), the nuclear pore number (*N*_p_) and length (*L*; Supplementary Table 1).


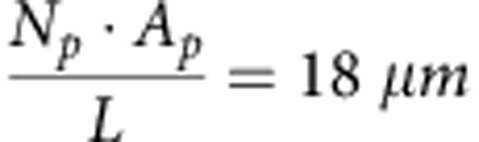



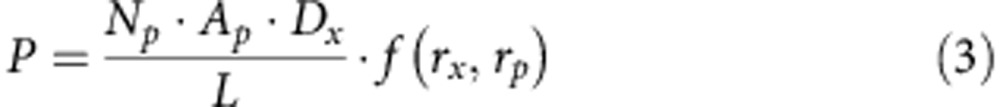


Finally, the rate of DARPP-32 active exportation outside the nucleus was obtained based on the concentration ratio between the nuclear and cytosolic DARPP-32 at equilibrium (equation (4)). The overall temporal profile of DARPP-32 nuclear translocation, both actively and passively, was validated by comparing with the experimental observations of how fast DARPP-32-GFP entered the nucleus after stimulating the photoactivable DARPP-32-GFP in the cytosol (Fig. 1h).


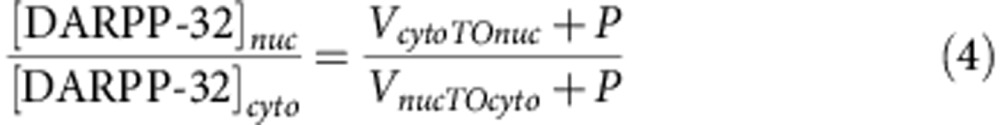


where *P* is the passive translocation rate and *V* is the active translocation rate.

To estimate PKA nuclear (nuc) translocation rate, the formulae from Renkin *et al*. (equation 3) was used^68^; taking into consideration the Stokes radius of PKA (*r_x_*=2.8 nm) and its diffusion coefficient (*D_x_*=4.4 μm^2^ s^−1^), as described above. This rate was validated by comparing the recovery of cytoplasmic (cyto) PKA subunits obtained from the simulation with experimental observations. The simulation was set up under the same conditions as described in ref. 65; that is adding forskolin to induce persistent cAMP elevation. As shown in Supplementary Fig. 3c, the translocation rate estimated was in agreement with the published experiments^65^.

### Statistics

Sample sizes were estimated from the expected effect size based on previous or preliminary experiments. No data was excluded, with the exception of obvious technical failure. Statistical analysis of experimental results were carried out using GraphPad Prism6 software. We utilized one-way analysis of variance and Tukey’s *post hoc* tests. *P* values <0.05 were considered significant. The coefficients of determination (*R*^2^) were calculated using software R for comparing numerical simulation results with experimental measurements.

## Author contributions

L.L. conceived, wrote and conducted the modelling work. L.L. and N.G. carried out live imaging experiments, analysed results and wrote the manuscript. J.-A.G. initiated, designed and supervised the study, analysed results and wrote the manuscript.

## Additional information

**How to cite this article:** Li, L. *et al*. Dendritic geometry shapes neuronal cAMP signalling to the nucleus. *Nat. Commun.* 6:6319 doi: 10.1038/ncomms7319 (2015).

## Supplementary Material

Supplementary Information Supplementary Figures 1-6, Supplementary Tables 1-6 and Supplementary References
