# Supplementary material for: Dendritic geometry shapes neuronal cAMP signalling to the nucleus
Source: Nat Commun. 2015 Feb 18;6:6319. doi: 10.1038/ncomms7319 (PMC4346624; doi:10.1038/ncomms7319)
Supplement: Supplementary Information — Supplementary Figures 1-6, Supplementary Tables 1-6 and Supplementary References [file ncomms7319-s1.pdf]

Supplementary Figures

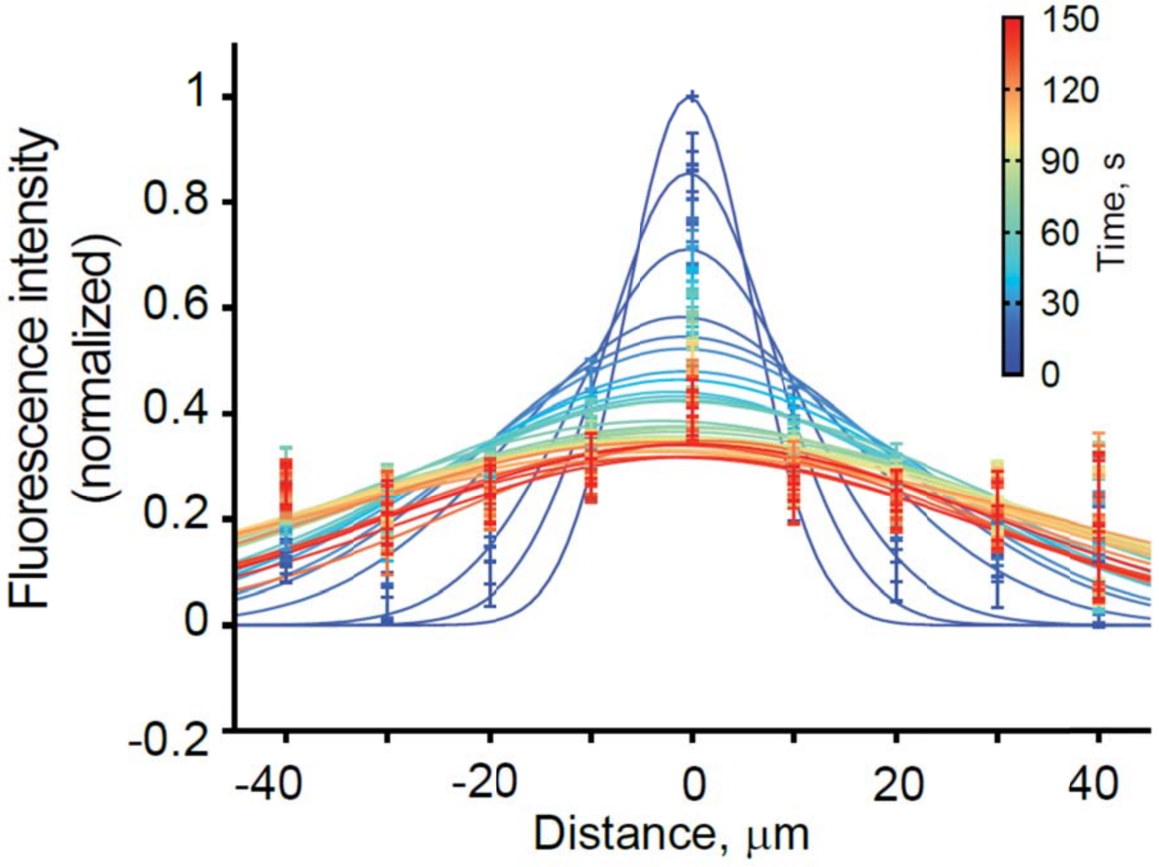

Supplementary Figure 1. DARPP-32 mobility in dendrites is not altered by stimulation of D1 dopamine receptors. Summary of mPA-GFP-DARPP-32 fluorescence spreading and decay in the dendrites of cultured striatal neurons, following photo-activation of the tag fluorescence (as in Fig. 1e), 15 minutes after dopamine D1 receptor activation (SKF81297, 10 µM). Data are means ± SEM of average fluorescence intensity for every 10-µm region (n = 7). Each color represents a time point, with 5-s intervals, for a total duration of 150 s.

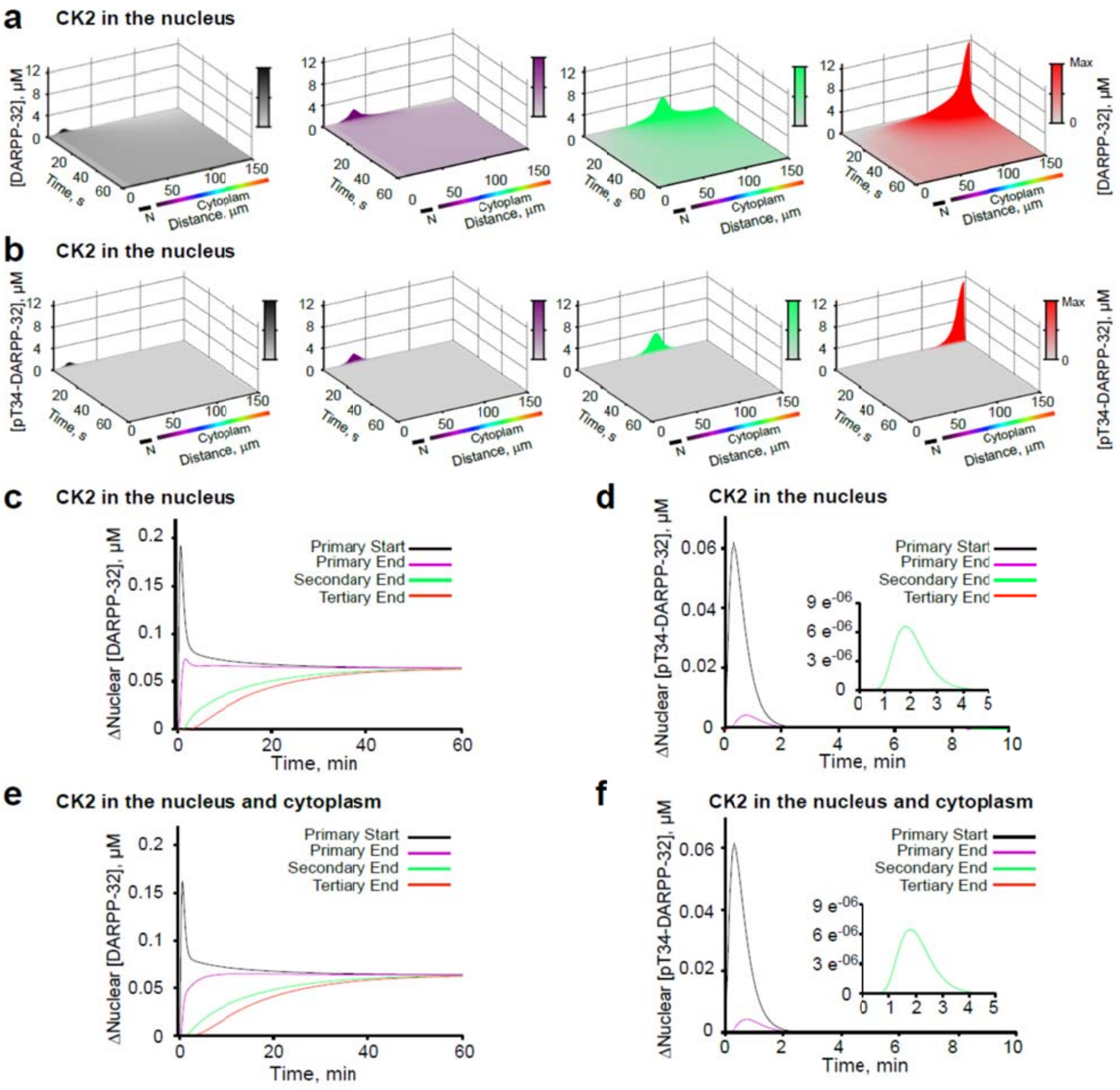

Supplementary Figure 2. Effects of CK2 subcellular localization on DARPP-32 or pT34-DARPP-32 diffusion. (**a, b**) Spatiotemporal changes in total DARPP-32 (**a**) or pT34-DARPP-32 (**b**) concentrations, after "release" of the same number of pT34-DARPP-32 molecules at the beginning of primary (black), end of primary (magenta), end of secondary (green), or end of tertiary dendrite (red), as in **Fig. 2b**, in the presence of CK2 in the nucleus. Time-course of DARPP-32 (**c**) or pT-DARPP-32 (**d**) average concentration in the nucleus, after release at 4 dendritic locations (as in **a, b**) in the presence of CK2 in the nucleus. (**e, f**) Time-course of DARPP-32 (**e**) or pT34-DARPP-32 (**f**) average concentration in the nucleus, after release at 4 dendritic locations in the presence of CK2 in both nucleus and cytoplasm.

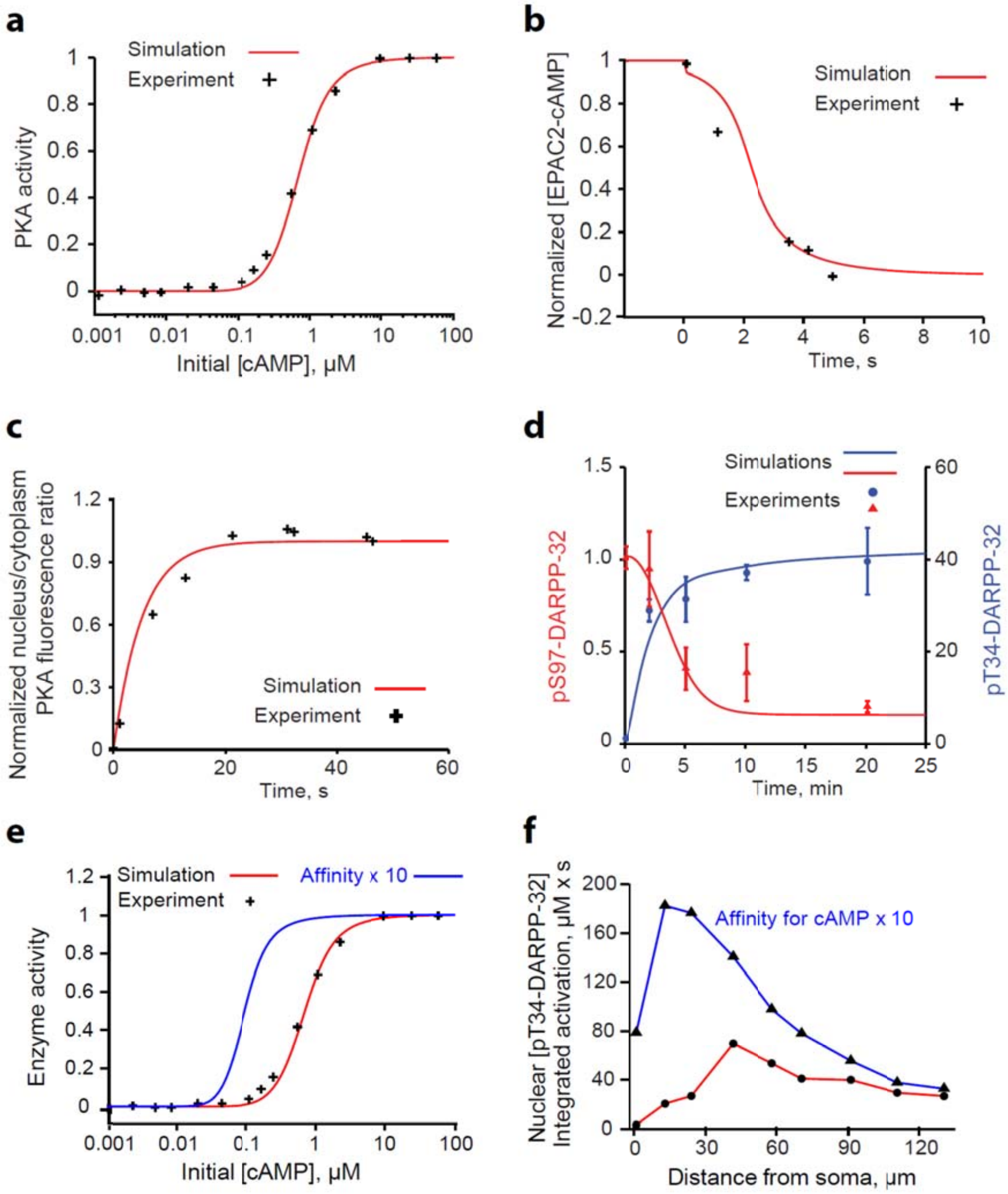

Supplementary Figure 3. Validation of model parameters. (**a-c**) Validation of the model parameters used for PKA activation (**a**), cAMP hydrolysis by PDE10A (**b**), and PKA nuclear translocation (**c**), by comparing model simulation results (red line) with published experimental data (black dots)[1-3]. Fit of published experimental data with our model: PKA activation (**a**), R^2 = 0.996; PDE catalytic ability (**b**), R^2=0.96; PKA nuclear translocation (**c**), R^2=0.98. (**d**) Validation of the model parameters used for phosphorylation and dephosphorylation of DARPP-32 at Thr34 (blue) and Ser97 (red), respectively, under persistent cAMP elevation, via comparison between published experimental data following forskolin treatment (points, means ± SEM)[4] and model simulation results (line). Fit of experimental data with model: pSer97, R^2=0.91; pThr34, R^2=0.94. (**e**) Effects of increasing 10-fold, in the model, the affinity of PKA regulatory subunit for cAMP on enzyme activity (blue). The same experimental points and simulation (red) as in (**a**) are shown for comparison. (**f**) Effects of increasing the affinity of PKA regulatory subunit for cAMP, on nuclear pT34-DARPP-32 nuclear activation as in Fig. 3e. Affinity as in Fig. 3e (red), 10-fold increased affinity (blue).

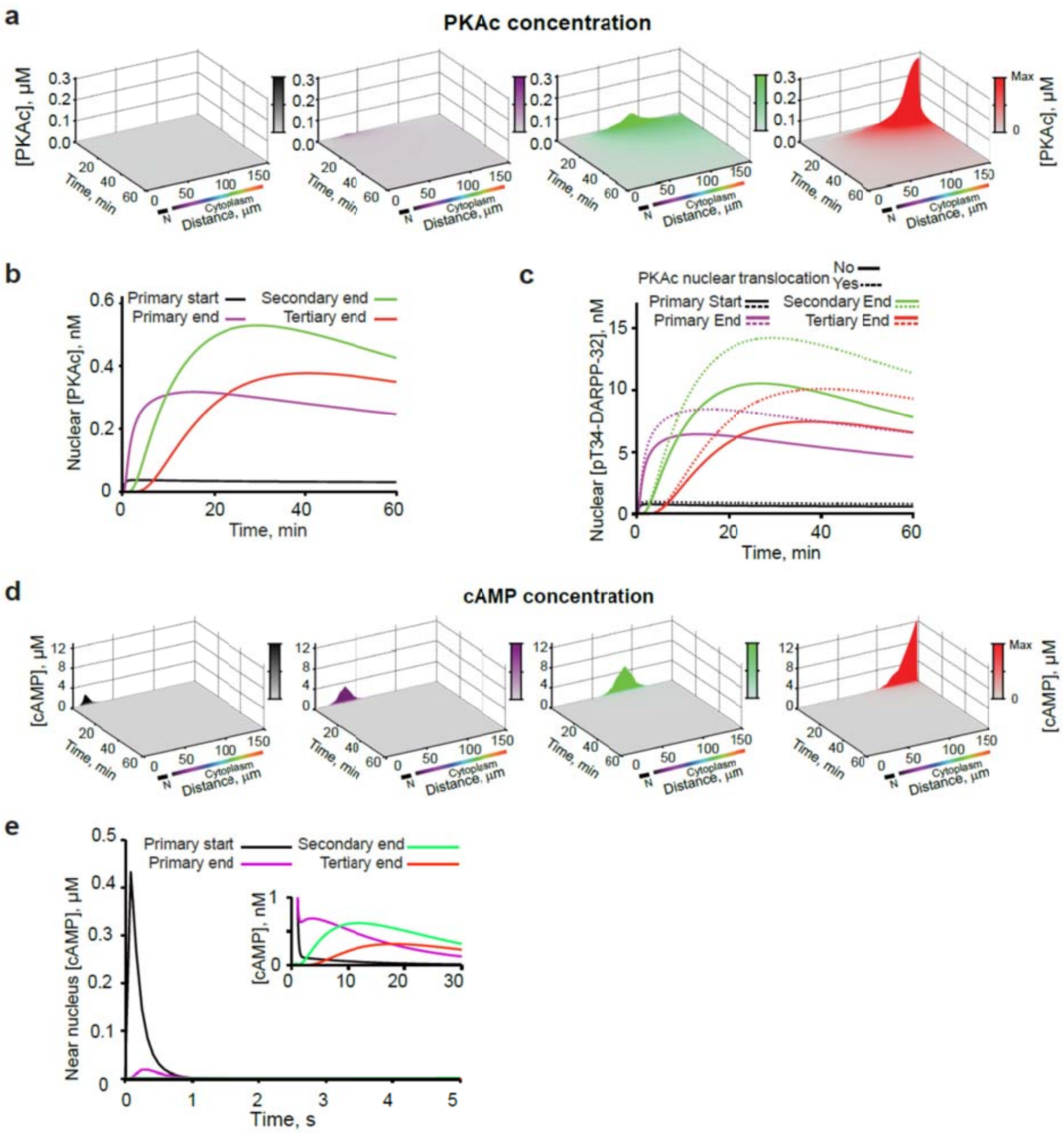

Supplementary Figure 4. Role of PKA and cAMP in the relationship between nuclear responses and distance from the site of dendritic stimulation. (**a**) Spatiotemporal evolution of PKA catalytic subunit concentration following local release of cAMP at 4 different locations as in **Fig. 3a**. (**b**) Time-course of nuclear catalytic PKA concentration after release of cAMP at 4 dendritic locations (black: beginning of primary; magenta: end of primary; green: end of secondary; red: end of tertiary dendrite). (**c**) In the same conditions as in b, time-course of nuclear pT34-DARPP-32 when nuclear translocation of PKA is blocked (solid lines). For comparison the modelling results in the absence of blockade is shown (dotted lines, same as in **Fig. 3d**). (**d**) Spatiotemporal evolution of cAMP concentration following local release of cAMP at 4 different locations. (**e**) Time-course of cAMP concentration in the vicinity of the nucleus as a function of time and of the site of cAMP release. Inset: same plots with different scales.

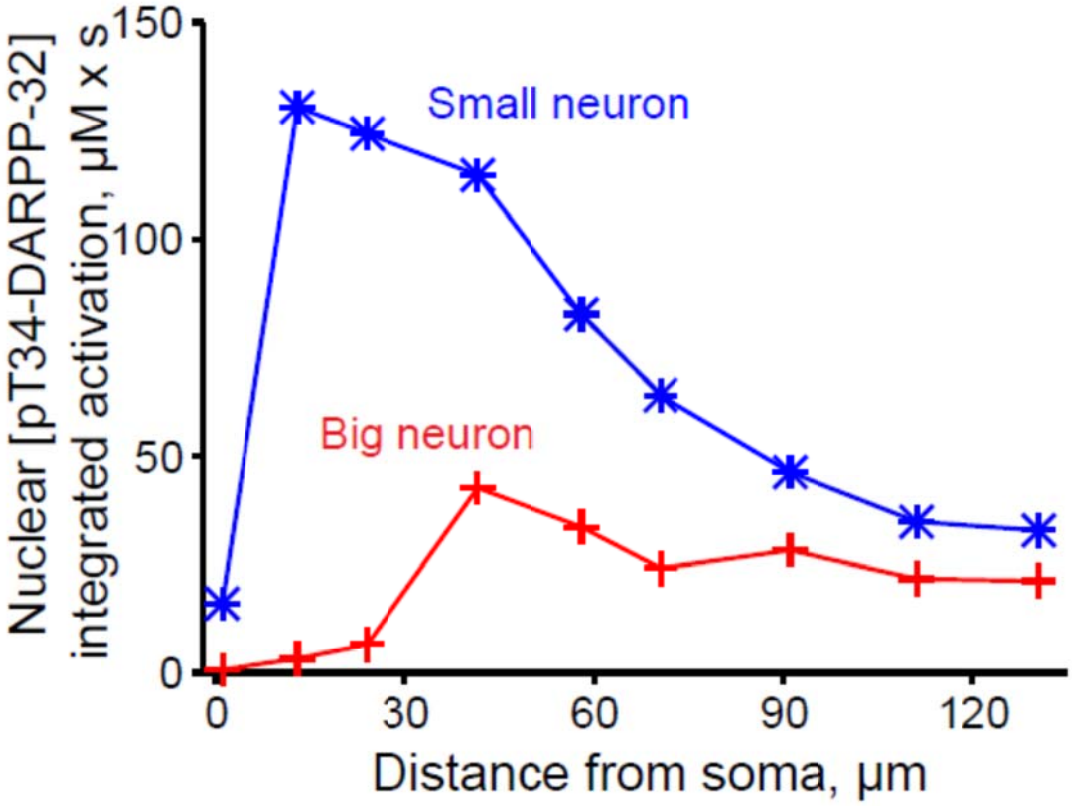

Supplementary Figure 5. Effects of maximal dendritic size on modeling results. Areas under the curve of nuclear pT34-DARPP-32 time-course following release of cAMP at 9 dendritic locations, as a function of the distance to soma, as in Fig. 3d. The shape of neurons were as in Fig. 2b, but the values for soma, nucleus and dendrites sizes used in this simulation were all the maximal or minimal values measured in the cultured striatal neurons used for FRET experiments (**Supplementary Table 1**): **Big neuron**: axes (diameters) of soma: 20.2, 12.6, 12.6 µm; diameter of nucleus, 10.4 µm; diameter of dendrites: primary, 3.2 µm; secondary, 2 µm; tertiary, 1.4 µm; **Small neuron**: axes (diameters) of soma: 15, 8, 8 µm; diameter of nucleus, 7 µm; diameter of dendrites: primary 2 µm, secondary 1.2 µm, tertiary 1 µm.

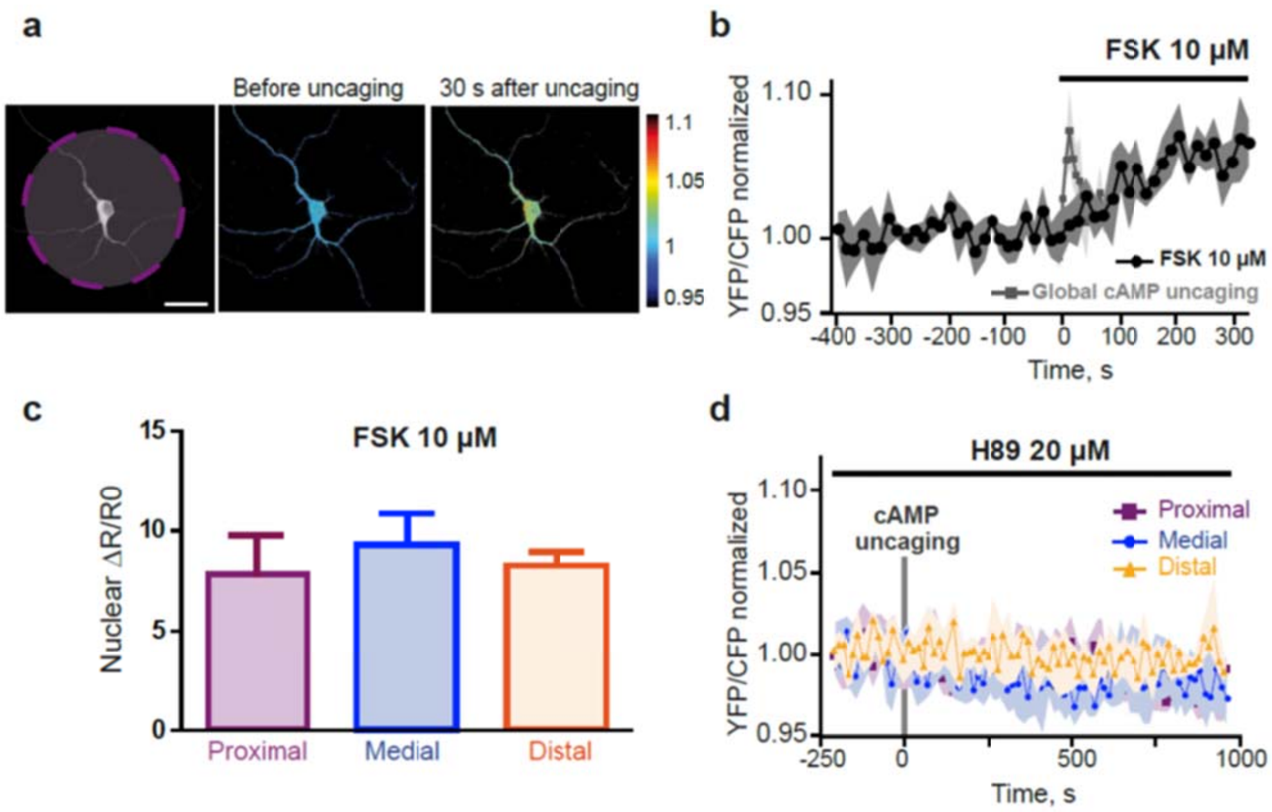

Supplementary Figure 6. Sensitivity and specificity of AKAR3 for FRET detection of PKA activation by cAMP uncaging in neurons. (**a**) Striatal neurons in culture were infected with Sindbis-AKAR3 and 6 h later loaded with DMNB-cAMP (caged cAMP). cAMP was released, as in **Fig. 5a** with a 1-s UV pulse covering the cell body and most of the dendrites (represented by the purple circle). Pseudo-color images represent the YFP/CFP fluorescence ratio pixel by pixel before and after cAMP uncaging. (**b**) Comparison of the time-course of normalized YFP / CFP fluorescence ratio of AKAR3 in the nucleus after **global cAMP uncaging** and bath application of 10 µM forskolin (**FSK**), which directly activates adenylyl-cyclase. Data are means ± SEM, n = 3. (**c**) At the end of localized stimulations in **Fig. 5b**, the responsiveness of the neuron was tested by adding 10 µM forskolin to the medium. Activation of AKAR3 in the nucleus was quantified as the increased YFP / CFP fluorescence ratio (ΔR) divided by the baseline fluorescence ratio (R0). No difference in the response to bath-applied forskolin was observed between the neurons previously stimulated in proximal, medial or distal dendrites, one-way ANOVA, $F(2,9) = 0.71$. (**d**) Localized dendritic cAMP uncaging was carried out as in Figure 3, in the presence of 20 µM H89, a PKA inhibitor and changes in PKA activity were detected in the nucleus as in **Fig. 5b**.

Supplementary Tables

Supplementary Table 1: Sources

	**References**
**Stokes radius and diffusion coefficients**	
DARPP-32	5
GFP	6-8
PKA catalytic subunit	9,10
Reverse relationship of diffusion coefficients with protein Stokes radius ratios	11
**Enzymatic reactions and binding**	
PDE10A	12
PKA	3
PKA interaction with kemptide	13
EPAC2 interaction with cAMP	14
Importin alpha interaction with its substrates	15
**Nuclear properties**	
Importin alpha concentration	16
Nuclear pore radius	17
Number of pores per nucleus	18
Transportation rate of GFP fused with NLS	15

Supplementary Table 2: Morphological characteristics of striatal neurons in culture

	n	Mean (µm)	Std. Deviation	Model neuron
Soma large axis (diameter)	27	17.58	2.544	20
Soma small axis (diameter)	27	10.35	2.294	10
Nucleus diameter	27	8.734	1.765	9
Primary dendrite diameter	27	2.682	0.5953	2.25
Secondary dendrite diameter	26	1.574	0.3034	1.75
Tertiary dendrite diameter	21	1.125	0.2128	1.25
Primary dendrite length	24	27.61	11.51	25
Secondary dendrite length	21	50.92	15.80	50
Tertiary dendrite length	6	58.50	9.793	60

Supplementary Table 3: Initial concentrations and diffusion coefficients

Species	Compartment	Initial Concentration (μM)	Diffusion Coefficient ($\mu m^2/s$)	References
cAMP	Cytosol	0	100	19,20
CK2	Nucleus	1	5	Estimated from 4
D97	Cytosol	50	7	Estimated from 4,21
D97	Nucleus	30	7	
PDE10A	Cytosol	1	5	22
PP1	Cytosol	5	4.6	23
PP1	Nucleus	5	4.6	
PP2A	Cytosol	2	3.6	24
PP2A	Nucleus	2	3.6	
PP2B	Cytosol	1.6	4.7	25
PP2B	Nucleus	1.6	4.7	
R2C2 (PKA holoenzyme)	Cytosol	1	2.7	26

Supplementary Table 4: Diffusion coefficients for other protein and complexes

Species	Diffusion Coefficients ($\mu m^2/s$)	Species	Diffusion Coefficients ($\mu m^2/s$)
cAMP_R2C2	2.7	D34_CK2	4.6
cAMP2_R2C2	2.7	D97	7
cAMP3_R2C2	2.7	D97_PKA	3.8
cAMP4_R2C2	2.7	D97_PP2Ap	3.4
cAMP4_R2	3.3	D	7
R2	3.3	D_PKA	3.8
R2C	3	D_CK2	5
cAMP4_R2C	3	PKA (catalytic subunit)	4.4
cAMP_PDE10A	5	PP1_D3497	4.3
D3497	7	PP1_D3497_PP2B	3.5
D3497_PP2A	3.4	PP1_D34	4.3
D3497_PP2B	4.3	PP1_D34_PP2B	3.5
D34	7	PP2A_PKA	2.5
D34_PP2B	4.3	PP2Ap	3.6

Supplementary Table 5: Reactions and parameters

Reaction	Compartment	Para-meter	Value	Reference
cAMP+PDE10A<=>cAMP_PDE10A	Cytosol	Kon	$80\ \mu M^{-1}.s^{-1}$	Estimated from 12
	Cytosol	Koff	$16.8\ \mu M^{-1}.s^{-1}$	
cAMP_PDE10A-> AMP+PDE10A	Cytosol	Kcat	$0.5\ s^{-1}$	
cAMP+R2C2<=>cAMP_R2C2	Cytosol	Kon	$54\ \mu M^{-1}.s^{-1}$	Estimated from 3
	Cytosol	Koff	$3\ s^{-1}$	
cAMP+cAMP_R2C2<=>cAMP2_R2C2	Cytosol	Kon	$54\ \mu M^{-1}.s^{-1}$	
	Cytosol	Koff	$3\ s^{-1}$	
cAMP+cAMP2_R2C2<=>cAMP3_R2C2	Cytosol	Kon	$75\ \mu M^{-1}.s^{-1}$	
	Cytosol	Koff	$110\ s^{-1}$	
cAMP+cAMP3_R2C2<=>cAMP4_R2C2	Cytosol	Kon	75 µM-1.s-1	
	Cytosol	Koff	110 s-1	
cAMP4_R2C2<=>cAMP4_R2C+PKA	Cytosol	Koff	0.1 s-1	
	Cytosol	Kon	$18\ \mu M^{-1}.s^{-1}$	
cAMP4_R2C<=>cAMP4_R2+PKA	Cytosol	Koff	$0.1\ s^{-1}$	
	Cytosol	Kon	$18\ \mu M^{-1}.s^{-1}$	
cAMP4_R2->R2+4 cAMP	Cytosol	Koff	$0.0012\ s^{-1}$	
R2C+PKA->R2C2	Cytosol	Kon	$18\ \mu M^{-1}.s^{-1}$	
R2+PKA->R2C	Cytosol	Kon	$18\ \mu M^{-1}.s^{-1}$	
PP2A+PKA<=>PP2A_PKA	Cytosol/Nucleus	Kon	$2.4\ \mu M^{-1}.s^{-1}$	27
	Cytosol/Nucleus	Koff	$0.1\ s^{-1}$	
PP2A_PKA->PP2Ap+PKA	Cytosol/Nucleus	Kcat	$0.3\ s^{-1}$	
PP2Ap->PP2A	Cytosol/Nucleus	Kf	$0.1\ s^{-1}$	Estimated
PKA+D<=>D_PKA	Cytosol/Nucleus	Kon	$5.6\ \mu M^{-1}.s^{-1}$	28
	Cytosol/Nucleus	Koff	$10.8\ s^{-1}$	
D_PKA->D34+PKA	Cytosol/Nucleus	Kcat	$2.7\ s^{-1}$	
D+CK2<=>D_CK2	Nucleus	Kon	$3.0\ \mu M^{-1}.s^{-1}$	29
	Nucleus	Koff	$10.0\ s^{-1}$	
D_CK2->D97+CK2	Nucleus	Kcat	$0.32\ s^{-1}$	
D34+CK2<=>D34_CK2	Nucleus	Kon	$3.0\ \mu M^{-1}s^{-1}$	
	Nucleus	Koff	$10.0\ s^{-1}$	
D34_CK2->D3497+CK2	Nucleus	Kcat	$0.32\ s^{-1}$	
D34+PP2B<=>D34_PP2B	Cytosol/Nucleus	Kon	$4.1\ \mu M^{-1}.s^{-1}$	30
	Cytosol/Nucleus	Koff	$6.4\ s^{-1}$	
D34_PP2B->D+PP2B	Cytosol/Nucldue	Kcat	$0.1\ s^{-1}$	

PP1+D34<=>PP1_D34	Cytosol/Nucleus	Kon	40 $\mu M^{-1}.s^{-1}$	31
	Cytosol/Nucleus	Koff	0.4 s^{-1}	
PP2B+PP1_D34<=>PP1_D34_PP2B	Cytosol/Nucleus	Kon	4.1 $\mu M^{-1}.s^{-1}$	30
	Cytosol/Nucleus	Koff	6.4 s^{-1}	
PP1_D34_PP2B->PP1+D+PP2B	Cytosol/Nucleus	Kcat	0.1 s^{-1}	
D97+PKA<=>D97_PKA	Cytosol/Nucleus	Kon	5.6 $\mu M^{-1}.s^{-1}$	28
	Cytosol/Nucleus	Koff	10.8 s^{-1}	
D97_PKA->D3497+PKA	Cytosol/Nucleus	Kcat	2.7 s^{-1}	
D97+PP2Ap<=>D97_PP2Ap	Cytosol/Nucleus	Kon	2.7 $\mu M^{-1}.s^{-1}$	Estimated from 4,32
	Cytosol/Nucleus	Koff	4.45 s^{-1}	
D97_PP2Ap->D+PP2Ap	Cytosol/Nucleus	Kcat	0.2 s^{-1}	
PP2B+D3497<=>D3497_PP2B	Cytosol/Nucleus	Kon	4.1 $\mu M^{-1}.s^{-1}$	30
	Cytosol/Nucleus	Koff	6.4 s^{-1}	
D3497_PP2B->D97+PP2B	Cytosol/Nucleus	Kcat	0.1 s^{-1}	
D3497+PP2Ap<=>D3497_PP2Ap	Cytosol/Nucleus	Kon	2.7 $\mu M^{-1}.s^{-1}$	Estimated from 4,32
	Cytosol/Nucleus	Koff	4.45 s^{-1}	
D3497_PP2Ap->D34+PP2Ap	Cytosol/Nucleus	Kcat	0.2 s^{-1}	
PP1+D3497<=>PP1	Cytosol/Nucleus	Kon	40 $\mu M^{-1}.s^{-1}$	31
	Cytosol/Nucleus	Koff	0.4 s^{-1}	
PP2B+PP1_D3497<=> PP1_D3497_PP2B	Cytosol/Nucleus	Kon	4.1 $\mu M^{-1}.s^{-1}$	30
	Cytosol/Nucleus	Koff	6.4 s^{-1}	
PP1_D3497_PP2B->PP1+D97+PP2B	Cytosol/Nucleus	Kcat	0.1 s^{-1}	

Supplementary Table 6: Nuclear translocation rates

	Species	Direction	Value (molecules.$\mu m^{-2}.\mu M^{-1}.s^{-1}$)
Passive diffusion	D	Both	1.62
	D34	Both	1.62
	D97	Both	1.62
	D3497	Both	1.62
	PKA (catalytic subunit)	Both	2.73
Active transportation	D	Cytosol To Nucleus	80
	D34	Cytosol To Nucleus	80
	D97	Cytosol To Nucleus	80
		Nucleus To Cytosol	134.43
	D3497	Cytosol To Nucleus	80
		Nucleus To Cytosol	134.43
	D_PKA	Cytosol To Nucleus	80
	D34_PP2B	Cytosol To Nucleus	80
	PP1_D34	Cytosol To Nucleus	80
	PP1_D34_PP2B	Cytosol To Nucleus	80
	D97_PKA	Cytosol To Nucleus	80
		Nucleus To Cytosol	134.43
	D97_PP2Ap	Cytosol To Nucleus	80
		Nucleus To Cytosol	134.43
	D3497_PP2B	Cytosol To Nucleus	80
		Nucleus To Cytosol	134.43
	D3497_PP2Ap	Cytosol To Nucleus	80
		Nucleus To Cytosol	134.43
	PP1_D3497	Cytosol To Nucleus	80
		Nucleus To Cytosol	134.43
	PP1_D3497_PP2B	Cytosol To Nucleus	80
		Nucleus To Cytosol	134.43

Supplementary References

1. Hagiwara, M., *et al.* Coupling of hormonal stimulation and transcription via the cyclic AMP-responsive factor CREB is rate limited by nuclear entry of protein kinase A. *Mol. Cell. Biol.* **13**, 4852-4859 (1993).
2. Nishi, A., *et al.* Distinct roles of PDE4 and PDE10A in the regulation of cAMP/PKA signaling in the striatum. *J. Neurosci.* **28**, 10460-10471 (2008).
3. Zawadzki, K.M. & Taylor, S.S. cAMP-dependent protein kinase regulatory subunit type IIbeta: active site mutations define an isoform-specific network for allosteric signaling by cAMP. *J. Biol. Chem.* **279**, 7029-7036 (2004).
4. Stipanovich, A., *et al.* A phosphatase cascade by which rewarding stimuli control nucleosomal response. *Nature* **453**, 879-884 (2008).
5. Hemmings, H.C., Jr., Nairn, A.C., Aswad, D.W. & Greengard, P. DARPP-32, a dopamine- and adenosine 3':5'-monophosphate- regulated phosphoprotein enriched in dopamine-innervated brain regions. II Purification and characterization of the phosphoprotein from bovine caudate nucleus. *J. Neurosci.* **4**, 99-110 (1984).
6. Slade, K.M., Baker, R., Chua, M., Thompson, N.L. & Pielak, G.J. Effects of recombinant protein expression on green fluorescent protein diffusion in Escherichia coli. *Biochemistry* **48**, 5083-5089 (2009).
7. Swaminathan, R., Hoang, C.P. & Verkman, A.S. Photobleaching recovery and anisotropy decay of green fluorescent protein GFP-S65T in solution and cells: cytoplasmic viscosity probed by green fluorescent protein translational and rotational diffusion. *Biophys. J.* **72**, 1900-1907 (1997).
8. Terry, B.R., Matthews, E.K. & Haseloff, J. Molecular characterisation of recombinant green fluorescent protein by fluorescence correlation microscopy. *Biochem. Biophys. Res. Commun.* **217**, 21-27 (1995).
9. Ochatt, C.M., Ulloa, R.M., Torres, H.N. & Tellez-Inon, M.T. Characterization of the catalytic subunit of Trypanosoma cruzi cyclic AMP-dependent protein kinase. *Mol. Biochem. Parasitol.* **57**, 73-81 (1993).
10. Zhong, H., *et al.* Subcellular dynamics of type II PKA in neurons. *Neuron* **62**, 363-374 (2009).
11. Young, M.E., Carroad, P.A. & Bell, R.L. Estimation of diffusion coefficients of proteins. *Biotech. Bioengin.* **22**, 947–955 (1980).
12. Nikolaev, V.O., Gambaryan, S., Engelhardt, S., Walter, U. & Lohse, M.J. Real-time monitoring of the PDE2 activity of live cells: hormone-stimulated cAMP hydrolysis is faster than hormone-stimulated cAMP synthesis. *J. Biol. Chem.* **280**, 1716-1719 (2005).
13. Denis, C.L., Kemp, B.E. & Zoller, M.J. Substrate specificities for yeast and mammalian cAMP-dependent protein kinases are similar but not identical. *J. Biol. Chem.* **266**, 17932-17935 (1991).
14. de Rooij, J., *et al.* Mechanism of regulation of the Epac family of cAMP-dependent RapGEFs. *J. Biol. Chem.* **275**, 20829-20836 (2000).
15. Bizzarri, R., Cardarelli, F., Serresi, M. & Beltram, F. Fluorescence recovery after

photobleaching reveals the biochemistry of nucleocytoplasmic exchange. *Anal. Bioanal. Chem.* **403**, 2339-2351 (2012).
16. Percipalle, P., Butler, P.J., Finch, J.T., Jans, D.A. & Rhodes, D. Nuclear localization signal recognition causes release of importin-alpha from aggregates in the cytosol. *J. Mol. Biol.* **292**, 263-273 (1999).
17. Keminer, O. & Peters, R. Permeability of single nuclear pores. *Biophys. J.* **77**, 217-228 (1999).
18. Ribbeck, K. & Gorlich, D. Kinetic analysis of translocation through nuclear pore complexes. *EMBO J.* **20**, 1320-1330 (2001).
19. Chen, C., Nakamura, T. & Koutalos, Y. Cyclic AMP diffusion coefficient in frog olfactory cilia. *Biophys. J.* **76**, 2861-2867 (1999).
20. Hempel, C.M., Vincent, P., Adams, S.R., Tsien, R.Y. & Selverston, A.I. Spatio-temporal dynamics of cyclic AMP signals in an intact neural circuit. *Nature* **384**, 166-169 (1996).
21. Halpain, S., Girault, J.A. & Greengard, P. Activation of NMDA receptors induces dephosphorylation of DARPP-32 in rat striatal slices. *Nature* **343**, 369-372 (1990).
22. Bhalla, U.S. & Iyengar, R. Emergent properties of networks of biological signaling pathways. *Science* **283**, 381-387 (1999).
23. Ingebritsen, T.S., Stewart, A.A. & Cohen, P. The protein phosphatases involved in cellular regulation. 6. Measurement of type-1 and type-2 protein phosphatases in extracts of mammalian tissues; an assessment of their physiological roles. *Eur. J. Biochem.* **132**, 297-307 (1983).
24. Fernandez, E., Schiappa, R., Girault, J.A. & Le Novere, N. DARPP-32 is a robust integrator of dopamine and glutamate signals. *PLoS Comput. Biol.* **2**, e176 (2006).
25. Goto, S., Matsukado, Y., Mihara, Y., Inoue, N. & Miyamoto, E. The distribution of calcineurin in rat brain by light and electron microscopic immunohistochemistry and enzyme-immunoassay. *Brain Res.* **397**, 161-172 (1986).
26. Hofmann, F., Bechtel, P.J. & Krebs, E.G. Concentrations of cyclic AMP-dependent protein kinase subunits in various tissues. *J. Biol. Chem.* **252**, 1441-1447 (1977).
27. Usui, H.*, et al.* Activation of protein phosphatase 2A by cAMP-dependent protein kinase-catalyzed phosphorylation of the 74-kDa B'' (delta) regulatory subunit in vitro and identification of the phosphorylation sites. *FEBS Lett.* **430**, 312-316 (1998).
28. Hemmings, H.C., Jr., Nairn, A.C. & Greengard, P. DARPP-32, a dopamine- and adenosine 3':5'-monophosphate-regulated neuronal phosphoprotein. II. Comparison of the kinetics of phosphorylation of DARPP-32 and phosphatase inhibitor 1. *J. Biol. Chem.* **259**, 14491-14497 (1984).
29. Girault, J.A., Hemmings, H.C., Jr., Williams, K.R., Nairn, A.C. & Greengard, P. Phosphorylation of DARPP-32, a dopamine- and cAMP-regulated phosphoprotein, by casein kinase II. *J. Biol. Chem.* **264**, 21748-21759 (1989).
30. King, M.M.*, et al.* Mammalian brain phosphoproteins as substrates for calcineurin. *J. Biol. Chem.* **259**, 8080-8083 (1984).
31. Hemmings, H.C., Jr., Greengard, P., Tung, H.Y.L. & Cohen, P. DARPP-32, a

dopamine-regulated neuronal phosphoprotein, is a potent inhibitor of protein phosphatase-1. *Nature* **310**, 503-505 (1984).
32. Hemmings, H.C., Jr., Nairn, A.C., Elliott, J.I. & Greengard, P. Synthetic peptide analogs of DARPP-32 (Mr 32,000 dopamine- and cAMP-regulated phosphoprotein), an inhibitor of protein phosphatase-1. Phosphorylation, dephosphorylation, and inhibitory activity. *J. Biol. Chem.* **265**, 20369-20376 (1990).
